# The Intersection of Machine Learning and Wireless Sensor Network Security for Cyber-Attack Detection: A Detailed Analysis

**DOI:** 10.3390/s24196377

**Published:** 2024-10-01

**Authors:** Tahesin Samira Delwar, Unal Aras, Sayak Mukhopadhyay, Akshay Kumar, Ujwala Kshirsagar, Yangwon Lee, Mangal Singh, Jee-Youl Ryu

**Affiliations:** 1 Department of Smart Robot Convergence and Application Engineering, Pukyong National University, Busan 48513, Republic of Korea; samira@pukyong.ac.kr (T.S.D.); aras2120@pukyong.ac.kr (U.A.); 2 Symbiosis Institute of Technology, Symbiosis International (Deemed University), Pune 412115, India; sayakpm3@gmail.com (S.M.); akshaykumarx17@gmail.com (A.K.); ujwala.kshirsagar@sitpune.edu.in (U.K.); 3 Department of Spatial Information Engineering, Pukyong National University, Busan 48513, Republic of Korea; modconfi@pknu.ac.kr

**Keywords:** Wireless Sensor Networks (WSNs), machine learning (ML), Quality of Service (QoS), Path Planning (PP), Sensor Node Deployment (SND)

## Abstract

This study provides a thorough examination of the important intersection of Wireless Sensor Networks (WSNs) with machine learning (ML) for improving security. WSNs play critical roles in a wide range of applications, but their inherent constraints create unique security challenges. To address these problems, numerous ML algorithms have been used to improve WSN security, with a special emphasis on their advantages and disadvantages. Notable difficulties include localisation, coverage, anomaly detection, congestion control, and Quality of Service (QoS), emphasising the need for innovation. This study provides insights into the beneficial potential of ML in bolstering WSN security through a comprehensive review of existing experiments. This study emphasises the need to use ML’s potential while expertly resolving subtle nuances to preserve the integrity and dependability of WSNs in the increasingly interconnected environment.

## 1. Introduction

The realisation of artificial intelligence (AI) across multiple fields is currently the focus of worldwide interest. Within this broader framework, Wireless Sensor Networks (WSNs) emerge as a strong facilitator, conspicuously exercising their effect across diverse industries such as healthcare facilities, smart infrastructure, precision agriculture, and industrial ecosystems [1].

Notably, the deployment of a large and dynamically dispersed array of sensor nodes is inevitably required for these expanding applications. As a result, they become the focal point of a complex web of rigorous limitations, including imperatives like minimising packet loss, extending network lifetime, increasing data throughput, optimising energy utilisation, and reducing transmission delays. As a result of this complex terrain, network managers are constantly faced with the onerous chore of fine-tuning a suite of stack settings to meet the rigorous expectations imposed by these many network situations.

The recent trajectory of technological development has been considerably altered by the convergence of micro-electro-mechanical system (MEMS) technology, mobile communications, and digital electronics. This convergence has ushered in a new age marked by the introduction of small but powerful sensor nodes with cost-efficiency, energy frugality, multifunctionality, and compact form factors. These sensor nodes, which include a slew of critical components such as the ability to sense, interpret data, and communicate wirelessly, serve as the foundation for the notion of sensor networks [2].

This paradigm change is dependent on the collaborative synergy generated by a slew of these small nodes, which pool their resources to create a disruptive technological environment. Sensor networks, in contrast to conventional sensors, which generally function in two independent deployment modes, result in a paradigm change with substantial implications.

- Conventional sensors are frequently limited to places far from the direct source of the phenomenon they aim to detect, relying on complicated procedures to separate the intended signal from the cacophony of ambient noise that surrounds it. To accomplish its discerning purpose, this strategy needs the deployment of considerably bigger sensors endowed with complicated signal processing capabilities.
- An alternate technique is the deployment of many sensors that are primarily concerned with perceiving the physical environment but lack comprehensive signal processing capabilities. To arrange both the physical location of these sensors and the extensive web of communication topologies that connects them in this situation, rigorous engineering is necessary. This complex interplay culminates in the transfer of time series data containing detected events to central nodes, where computational alchemy occurs and data fusion transforms raw data into useful insights. This contrast between the old sensor paradigm and the transformational sensor network ethos emphasises the latter’s seismic shift in strategy and technical innovation.

### Research Gap

A sensor network is a collection of densely distributed sensor nodes that may be put at random in difficult or inaccessible terrain, allowing nodes to self-organise and cooperate. This allows for localised calculations, delivering only essential, partially processed data [3]. These qualities broaden the scope of sensor network applications, such as healthcare, military, and security. WSN technology has various advantages over traditional networking systems, including cheaper costs, scalability, reliability, accuracy, flexibility, and rapid installation, making it essential in a variety of applications [4].

Beyond explicit programming, machine learning (ML) is a transformative process that learns and adapts on its own by experiential learning [5]. Because of its interdisciplinary nature, machine learning is useful in a variety of fields, including engineering, medicine, and computer science. Recent advances in machine learning (ML) applications have offered novel ways for deploying WSNs [6]. ML serves as a foundation for the integration of many technical domains, seamlessly linking cyber-physical infrastructures, the Internet of Things (IoT), and machine-to-machine (M2M) interactions [6]. Traditionally, deployments have depended on entrenched traditions acquired from practical experience and heuristic guidance; however, there is an urgent need for a more precise method for discovering and codifying patterns, hence reducing the knowledge gap. An ideal approach to pursuing an astute configuration strategy for WSNs should include a structured workflow that includes five essential components: problem elucidation, meticulous data collection, rigorous data analysis, insightful data visualisation, and methodical evaluation, using machine learning techniques such as support vector machines, logistic regression, and random forests [7,8].

WSNs have made great advances in a variety of fields, but a key research gap still exists at the junction of WSN security and machine learning (ML) for cyber-attack detection. WSNs’ particular restrictions, such as low processing capacity, energy limits, and decentralised architecture, make it difficult to include strong security methods. Although several machine learning techniques have been used to improve WSN security, many previous studies do not address the scalability, flexibility, and real-time response necessary for dynamic settings and changing threat landscapes.

This review paper aims to provide an in-depth analysis of the current intersection between machine learning and WSN security, with a specific focus on cyber-attack detection, evaluating the effectiveness of various machine learning models and approaches used in WSNs, identifying key limitations, and highlighting potential areas for future research, synthesising existing studies to uncover gaps in the literature and proposing directions for optimising security models in WSN.

However, as the Internet of Things (IoT) environment grows, standard machine learning algorithms fall short in terms of effective feature extraction [9]. Table 1 shows a summary of some research papers. Indeed, the vast majority of classical machine learning algorithms used in IoT systems have shallow structures with limited modelling and representational capabilities. As a result, there is an urgent need to embrace a deep learning paradigm that promises to uncover nuanced patterns hidden within the quantity of raw data common in various IoT applications. Deep learning architectures, strong frameworks, and specialised hardware developments have elevated deep learning to the preeminent alternative for the deployment of very complicated and potent models.

**Figure 1:**
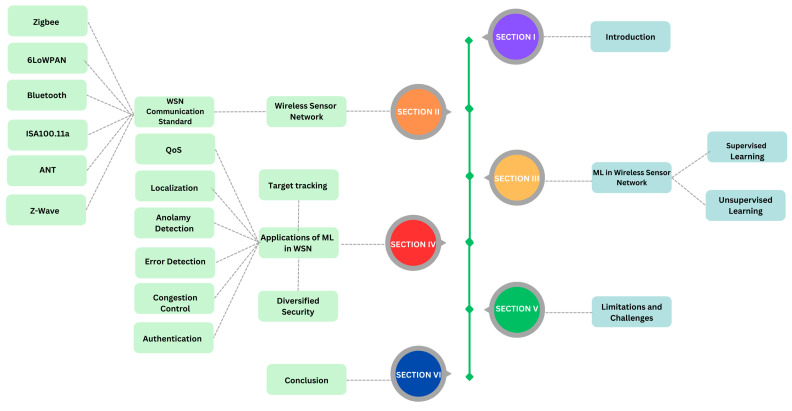
Taxonomy of the review.

Figure 1 depicts the interaction of several components of a WSN architecture, including the processes of Sensor Node Deployment (SND), Path Planning (PP), and Quality of Service (QoS) management. Sensor Node Deployment (SND) refers to the deliberate deployment of sensor nodes throughout the network to achieve optimal coverage and connection. Once the nodes have been installed, Path Planning (PP) is used to establish the most effective data transmission paths, taking into consideration network restrictions like energy efficiency and transmission latency.

To ensure peak performance, the network’s Quality of Service (QoS) is monitored and adjusted. This involves maintaining consistent data transfer, lowering latency, and increasing total throughput. Machine learning (ML) approaches are used to improve several elements of the WSN, such as security, error detection, and traffic prediction. Furthermore, the Medium Access Control (MAC) protocol governs how data packets are transferred throughout the network, eliminating data collisions and maintaining seamless communication between nodes. These components work together to provide a strong and efficient wireless network that can adapt to changing environmental conditions and operating requirements.

**Figure 2:**
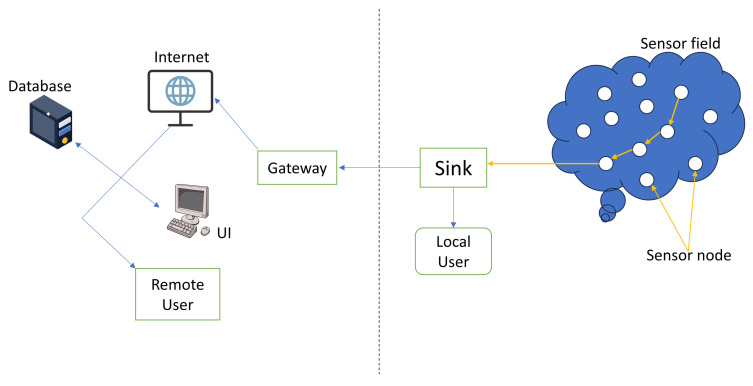
Basic configuration of a WSN architecture.

Figure 2 illustrates the standard configuration of a basic WSN.

The remainder of the paper is described as follows: The introduction and applications of WSN are covered in Section 2. Section 3 delves into the ML techniques used in WSN. Section 4 discusses machine learning applications in WSN, whereas Section 5 looks at WSN limitations and issues. The paper concludes with Section 6.

## 2. Wireless Sensor Networks

This section introduces the notion of WSNs, a crucial technology used in a variety of applications such as healthcare, environmental monitoring, and industrial systems. This section will give an overview of the WSN architecture, main components, and their responsibilities in data collecting and communication. We will also look at the inherent issues that WSNs confront, such as scalability, energy efficiency, and data accuracy.

A WSN is a complex network of tiny sensor nodes strategically deployed throughout a geographical area, creating a harmonic symphony of cooperative data exchange via wireless conduits. These sensor nodes act as diligent data collectors inside the monitored landscape, reporting their results to a centralised repository known as a sink. This sink, in turn, is responsible for data processing, either locally or by connections with larger networks, including the enormous expanse of the Internet, supported by gateways [4].

A typical sensor node or mote, which is the fundamental building element of a WSN, is a multidimensional entity that includes a variety of critical components. These nodes house processors that manage mote operations and perform data processing functions. Sensors attached to the device can detect environmental characteristics such as temperature, humidity, and light. However, it is critical to recognise that these motes function under rigorous limits, such as limited computing capacity and limited sensing capabilities due to bandwidth and battery constraints [18].

Memory resources are critical for storing programme instructions as well as the wide range of sensor data, from raw measurements to processed insights. The motes communicate with one another using a low-rate (10–100 kbps) and short-range (less than 100 m) wireless radio, which is commonly based on IEEE 802.15.4 radio standards. Given wireless communication’s ravenous thirst for power, effective energy-conscious communication strategies are required to maintain mote operation. Rechargeable batteries develop as a widespread power source in this environment. Motes contain energy-harvesting technologies like solar cells to enhance their durability in distant and harsh situations, allowing for years of unattended deployment [19].

A WSN’s Sensor Node Deployment may be divided into two paradigms: ad hoc and preplanned. Ad hoc installations are useful in large, open areas where a large number of nodes may be spread for autonomous monitoring and reporting. However, because of the vast number of nodes involved, this strategy complicates network maintenance and failure detection. Preplanned deployments, on the other hand, are appropriate for scenarios requiring restricted coverage, where a prudent selection of nodes is strategically positioned, resulting in lower network maintenance and administration costs [20].

A WSN is a complex ecosystem made up of multiple sensor nodes engaged in intricate inter-node communication over a wide range of radio frequencies, capable of performing a variety of critical tasks such as sensing, surveillance, measurement, and tracking [10]. These wireless nodes are absolutely resource-bound while being essential to their tasks, as seen by their confined processor power, bandwidth, battery life, and memory capacity [11].

**Figure 3:**
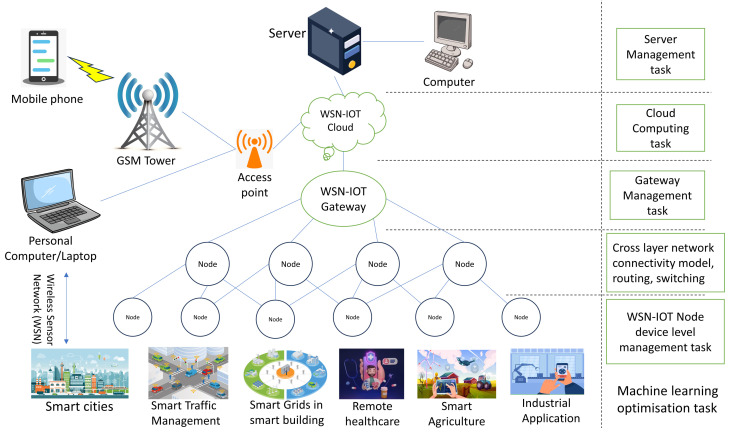
Complex WSN flow.

As seen in Figure 3, the complicated network of communication within the levels of a WSN is a complex ballet. The network architecture is shaped by WSNs, and the perception layer routing tables are meticulously updated by them. To do this, a diversified set of protocols is used to methodically manage the sophisticated network infrastructure [21]. Following that, the WSN begins the critical role of data acquisition, gathering information from multiple geographical vantage points and sending it to the network layer, also known as the “edge router”. It is critical to recognise that WSN nodes, these network layers’ fundamental building blocks, differ from conventional wireless networks in a number of ways [22]. These distinctive qualities include the following.

- Decentralisation: WSN nodes function autonomously without the governance of a central controller.
- Stationarity and mobility: These nodes can operate as either stationary fixtures or mobile entities, bringing dynamism to the network’s composition.
- Restricted transmission range: The propagation distance of WSN nodes is intrinsically restricted, which contributes to the complicated structure of their communication.
- Dynamic network topology: The network topology of the WSN is constantly changing, necessitating adaptive communication solutions.
- Multi-hop connectivity: Data transmission frequently involves multiple-hop connections, adding to the complexity of network communication.
- Bandwidth limitations: As bandwidth resources are limited, careful data management and transmission are required.

Despite their critical duties, WSN nodes work in potentially unsafe conditions with infrequent monitoring. This intrinsic weakness highlights the lingering possibility of sensitive data leaking to unauthorised parties, resulting in serious security and privacy issues [23]. Mitigating these risks is critical if WSNs are to reach their full potential in a safe and reliable way.

There are two prominent protocols in the field of WSNs, ZigBee and 6LoWPAN, which play critical roles in supporting network administration inside the perception layer. These protocols, on the other hand, have exceptional versatility that goes beyond their basic areas, including a wide range of network media. This adaptability includes support for low-power Wi-Fi technologies, easy integration with Bluetooth communication standards, and participation in sub-1 GHz radio frequencies [21].

This section has presented a thorough overview of WSNs, emphasising their design, applications, and problems. Notable aspects include the importance of efficient sensor node placement and the requirement for appropriate energy management systems to maximise network performance. The following section will delve deeper into the standards that govern Communication Protocols in WSNs, laying the groundwork for improved data transmission efficiency and security.

### 2.1. WSNs Communication Standard

This section analyzes the communication standards that are critical to the efficient operation of WSNs. It will investigate essential protocols such as IEEE 802.15.4, ZigBee, and Bluetooth, among others, and their effects on data transfer, network organisation, and energy efficiency. A detailed grasp of these standards is required to optimise WSN performance and ensure smooth communication between sensor nodes.

**Figure 4:**
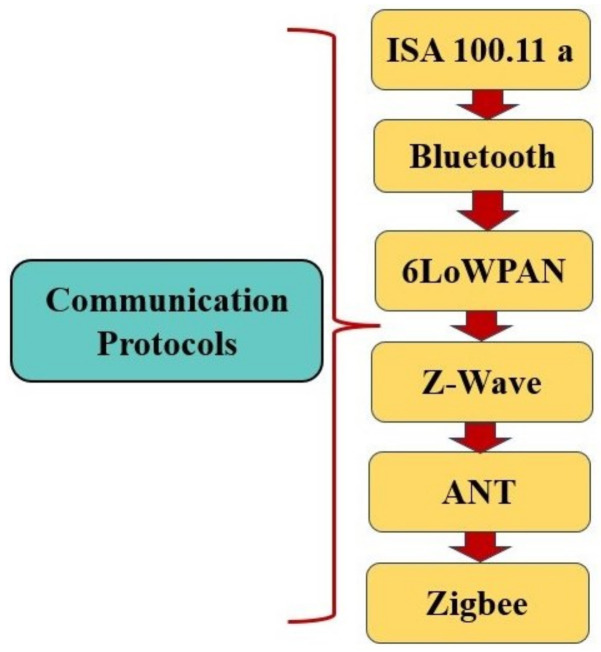
Illustration of different wireless communication standards for WSNs.

The IEEE 802.15.4 standard emerges as the definitive standard controlling data communication devices that operate within the constraints of Low Rate Wireless Personal Area Networks (LR-WPANs), methodically created by the IEEE 802.15.4 Working Group. It presents a compelling offering distinguished by cost-efficiency, limited range, low power consumption, and a conservative data transmission rate, therefore responding to the nuanced needs of sensor networks. This standard, designed specifically for wireless sensor applications, prioritises short-range communication as a strategic manoeuvre to maximise battery longevity [13]. Figure 4 represents the different Communication Protocols in WSNs.

**Figure 5:**
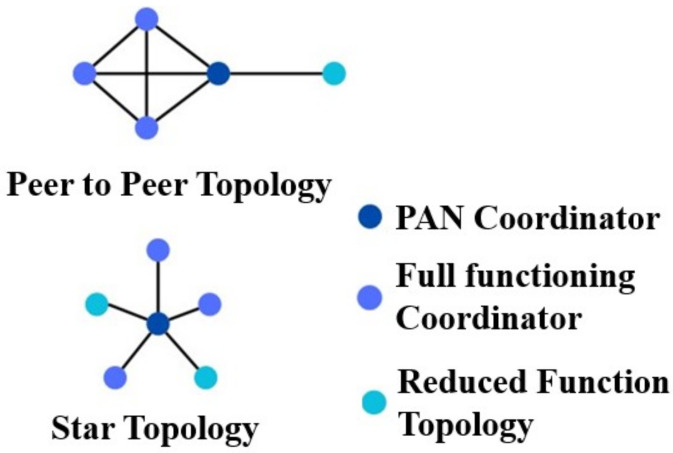
Topologies of Communication Protocols in WSNs.

In delving deeper into the operational aspects of IEEE 802.15.4, it is critical to highlight its support for two alternative network topologies: the star and peer-to-peer configurations, both of which exemplify the standard’s versatility, as seen in Figure 5.

The star topology focuses on a central controller (Full Function Device or FFD) that orchestrates communication across all network devices [12]. In contrast, peer-to-peer topology ushers in a more complex network environment, allowing for advanced forms such as mesh networking architecture. Networks can dynamically emerge in an ad hoc way inside this peer-to-peer paradigm, with inherent self-organisation and self-healing capabilities. The decision between star and peer-to-peer topologies is influenced by crucial parameters such as coverage area and latency. The star topology, with its low latency and appropriateness for smaller coverage zones, fits nicely with applications that require quick response. The peer-to-peer topology, on the other hand, excels in broad coverage zones where latency is unimportant and the network thrives on adaptability and scalability. Table 2 compares the features of different Communication Protocols.

#### 2.1.1. ZigBee

ZigBee, a prominent player in the field of wireless communication technologies, is a reliable option distinguished by its low cost and energy consumption, and it is frequently used in embedded systems. This strong standard is meticulously developed under the watchful eye of the ZigBee Alliance [27]. Its main strength is the provision of mesh networking capabilities to 802.15.4 applications, which represents a big step forward in the evolution of wireless communication.

ZigBee deployment often entails the addition of auxiliary parts like a coordinator and a router in addition to the fundamental ZigBee end device. This architectural complexity guarantees that ZigBee networks run smoothly. Notably, a traditional ZigBee node integrated into a wider network ecosystem needs the presence of an 802.15.4/IP gateway to enable communication with IP-centric devices. Therefore, applications for WSNs that do not involve interacting with IP-enabled devices are the ideal places to use ZigBee’s features.

#### 2.1.2. 6LoWPAN

The 6LoWPAN standard was cleverly included in RFC 4944 by the Internet Engineering Task Force (IETF) [24], ushering in a transformative era by coordinating IPv6 communication on top of the intricate structure of IEEE 802.15.4 networks. “IPv6 over Low-power Wireless Personal Area Networks”, or 6LoWPAN, is a paradigm shift that promotes IPv6 packet transmission over IEEE 802.15.4 networks with low power and low data rates. This significant accomplishment provides a helping hand to interoperability, allowing for smooth integration with a wide range of IP-enabled devices. The distinguishing feature of 6LoWPAN is its ability to permit direct communication between 6LoWPAN devices and their IP-enabled equivalents, hence eliminating the borders that may have previously compartmentalised these devices. The IP for Smart Objects (IPSO) Alliance, which is a significant supporter, ardently advocates for the adoption of integrated IP technologies and 6LoWPAN in the area of smart devices. 6LoWPAN enables unprecedented possibilities by enclosing these devices within the huge ecosystem of IP communication and control.

Considering the significant difference in packet sizes between IPv6 and the more restrictive IEEE 802.15.4 frame dimensions, the creative inclusion of an adaption layer, tucked ingeniously between the MAC (Medium Access Control) layer and the network layer, becomes a need. This crucial layer acts as the pivot for optimisation, effectively connecting IPv6 with IEEE 802.15.4. The adaption layer orchestrates the art of IPv6 packet header compression within its domain, as well as the expert management of fragmentation and the smooth reassembly of pieces. These harmonic mechanisms not only allow IPv6 packets to be sent via IEEE 802.15.4 networks, but they also open the path for the convergence of two distinct communication paradigms.

#### 2.1.3. Bluetooth

Bluetooth, a wireless technology designed for close-range communication between low-cost devices, has emerged as a strong cable-replacement alternative in Wireless Personal Area Networks (WPANs). Its operating canvas unfolds in the 2.45 GHz ISM (Industrial, Scientific, and Medical) band, where it skillfully utilises frequency hopping techniques to battle interference and decrease signal fading, providing reliable communication. Bluetooth’s communication radius is from 10 to 100 m, and it offers a respectable data transfer rate of up to 3 Mbps. It is worth noting that this standard, which was initially established under the auspices of IEEE 802.15.1, has progressed beyond the bounds of traditional maintenance. The Bluetooth Special Interest Group currently has control over Bluetooth, and it made a significant advancement in 2010 by releasing the Bluetooth Core Specification Version 4.0.

As we go on to Bluetooth v4.0 [25], we see the addition of Bluetooth Low Energy (BLE) technology [28], a ground-breaking development that allows a new generation of Bluetooth smart devices to function with unmatched efficiency, drawing power from small coin-cell batteries and sustaining operational vitality for months or even years. Numerous fascinating applications, such as those in medical care, fitness and sports, security, and household entertainment, have been made possible by this game-changing technology.

#### 2.1.4. ISA100.11a

The ISA100.11a standard [29], developed by the ISA100 standards committee, an affiliate of the prestigious International Society of Automation (ISA), is a sturdy symbol of innovation in the field of industrial automation. This standard, precisely tailored to meet the stringent standards of industrial applications, extends its welcoming arms to embrace a variety of network topologies, ranging from the simple star configuration to the complex mesh networking architecture. We encounter a trinity of important components inside the ecosystem of an ISA100.11a WSNs: field devices, gateways, and handheld devices, each playing a crucial part in orchestrating the symphony of industrial data transfer.

The exceptionally low latency of ISA100.11a, which results in a lightning-quick response time of 100 milliseconds, is a distinguishing characteristic that sets it apart. In the complex dance of frequency management, ISA100.11a applies the 2.4 GHz ISM (Industrial, Scientific, and Medical) band exclusively. This strategic decision is strengthened further by the prudent application of frequency hopping methods, a smart manoeuvre aimed to improve dependability while creating sturdy walls against interference from neighbouring wireless networks. The combination of these characteristics solidifies ISA100.11a as a staunch standard in the realm of industrial automation, where accuracy and efficiency reign supreme.

#### 2.1.5. ANT

ANT [14], an enigma in the world of wireless communication, provides a wireless Communication Protocol stack that reveals its unique identity as a proprietary technology and locates itself in the field of ultra-low power networking applications. Its core resides in its ability to work smoothly inside the embrace of low-cost, low-power microcontrollers and transceivers, all of which are put in motion within the teeming ecology of the 2.4 GHz ISM band. One distinguishing feature of ANT’s attraction is its adaptability, as seen by its ability to accommodate varied network topologies.

These configurations range from simple peer-to-peer arrangements to the complicated tapestry of star, tree, and other kinds of mesh networking, all of which are contained inside Personal Area Networks (PANs). ANT is the keystone in this picture, perfectly positioned to cater to the domains of sports, fitness, wellness, and home health applications, where the combination of technology and human well-being takes centre stage. The admirable data rate it provides, a towering 1 Mbps, easily outperforms the capabilities of its IEEE 802.15.4 equivalent, which trundles along at a comparably modest 250 kbps, is a credit to its energy-efficient architecture.

#### 2.1.6. Z-Wave

Z-Wave, an example of a low-powered RF-based wireless communication technology, emerges as a one-of-a-kind solution geared to the realm of remote control applications, with a favourable eye on residential and light commercial domains. Zensys conceived and fostered it, but it is now safely in the hands of the Z-Wave Alliance [26]. The distinguishing feature that sets Z-Wave apart from its competitors, notably IEEE 802.15.4-based technology, is its purposeful choice to operate in the sub-1 GHz range, snuggled securely around the 900 MHz spectrum.

Mesh networking is intricately woven into the fabric of Z-Wave technology, a critical element that enhances its stability and durability. The available data speeds of 9.6 kbps and 40 kbps, along with an exceptional maximum outdoor range of 30 m, solidify its place as a powerful contender in the wireless communication area. Z-Wave’s beautiful tone reverberates across home and light business settings, where accuracy and robustness are vital.

This section has analyzed the essential communication standards that underpin the functionality of WSNs, highlighting the contributions of each protocol to data flow management and network stability. Building on this foundation, the focus will now shift to the practical applications and types of WSNs, examining their deployment across diverse industries and use cases.

### 2.2. Types of WSNs

**Figure 6:**
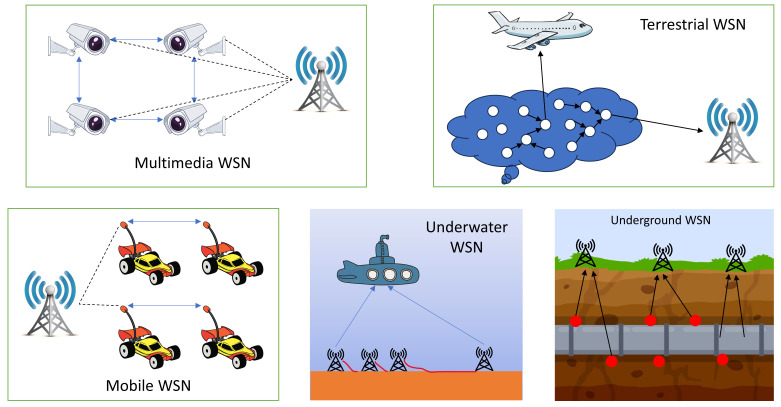
Overview of various deployment types in WSNs.

WSNs are currently being used in a wide range of terrains, including terrestrial landscapes, subterranean depths, and aquatic domains. In order to shed light on this multifaceted tapestry of WSN deployments, we propose a taxonomy that divides these networks into five distinct categories, each with its own set of complexities [30], a visual representation of which is thoughtfully illustrated in Figure 6, serving as a navigational compass through the complex terrain of WSN deployment scenarios.

- Terrestrial WSNs are systems of dozens to thousands of inexpensive nodes that are placed strategically all over the world’s surface. These nodes are generally deployed haphazardly, using means like aerial drops from aeroplanes. The challenge with terrestrial WSNs [31] is to provide smooth data connection back to the central base station in the midst of a densely populated area.
  Because of the inherent limitations of finite, non-rechargeable battery power, terrestrial sensor nodes usually include supplemental power sources like solar cells. Effective multihop routing, constrained transmission range, in-network data aggregation, and the utilisation of low-duty-cycle operations all contribute to energy savings. Environmental sensing, industrial monitoring, and terrestrial exploration are just a few of the uses for terrestrial WSNs.
- Underground Wireless Sensor Networks (UGWSNs) are network configurations in which sensor nodes are deliberately placed in underground settings such as caverns, mines, or beneath the Earth’s surface [32]. To help with data transmission from underwater sensor nodes to the top layer, additional sink nodes are positioned above the surface. These networks are often more expensive than terrestrial versions, owing to the need for specialised equipment to provide reliable communication through liquid, stone, and impurities.
  Given these conditions, signal attenuation and loss make wireless communication problematic. The subterranean placement of nodes makes replenishing or charging their batteries difficult, necessitating the development of energy-efficient Communication Protocols for extended operational life. UGWSNs applications include agricultural monitoring, landscape management, subsurface soil, water, and mineral monitoring, and military border surveillance.
- Underwater Wireless Sensor Networks (UWWSNs) comprise a set of sensors positioned below the water’s surface, notably in marine environments [33]. Only a small number of nodes are installed because of the high node costs, and autonomous submersibles are used for exploration and data collection. Acoustic signals, serving as the fundamental medium for underwater wireless communication, face a variety of challenges, including constrained capacity, extended propagation delays, increased latency, and signal loss issues. To survive in the severe circumstances of the maritime world, these nodes must be capable of self-configuration and adaptation. Energy-efficient underwater interaction and collaboration strategies are essential since underwater nodes have finite, non-rechargeable battery lives. Technologies of UWWSNs include equipment surveillance, disaster prevention and tracking, tracking of seismic activity, underwater robotics, and the monitoring of pollution.
- Multimedia Wireless Sensor Networks (MUWSNs) are made up of low-cost sensor nodes that are armed with a variety of sensors, including cameras and microphones, and are strategically placed in advance to provide complete coverage [34]. Video, audio, and picture data types may all be stored, processed, and retrieved using multimedia sensor devices. They deal with a number of challenges, such as increasing bandwidth needs, greater use of energy, provisioning for Quality of Service (QoS), data processing, and approaches to compression as well as cross-layer design. The transmission methods designed for multimedia WSNs must strike a compromise between meeting high bandwidth needs and consuming little energy, especially when delivering multimedia material such as video streams. Although attaining constant QoS in multimedia WSNs remains difficult owing to changing connection capacity and latency, achieving a certain degree of QoS is critical for dependable content delivery. These networks supplement existing WSN applications, notably in the tracking and monitoring domains.
- Mobile Wireless Sensor Networks (MOWSNs) have sensor nodes that are mobile, allowing them to travel and interact with their actual environment [15]. These mobile nodes may move across the network and self-organise, integrating sensing, computing, and communication capabilities. Mobile WSNs, as opposed to static WSNs that rely on fixed routing, require dynamic routing methods. Mobility poses a variety of challenges, including those related to deployment strategies, managing mobile nodes, real-time positioning, guiding and supervising moving elements, ensuring adequate sensing coverage, consuming less energy while moving, maintaining network connectivity, and optimising data dissemination. Mobile WSNs’ primary uses include environmental monitoring, habitat surveillance, undersea exploration, military reconnaissance, target tracking, and search-and-rescue operations. When compared to static networks, these networks have the ability to provide better coverage and connection.

### 2.3. Applications of WSNs

This section delves into the diverse uses of WSNs in healthcare, industrial automation, environmental monitoring, and home automation. WSNs have become critical in various industries, with each application being adapted to meet its individual requirements. This presentation will focus on the technology’s adaptability and scalability in responding to varied use cases.

**Figure 7:**
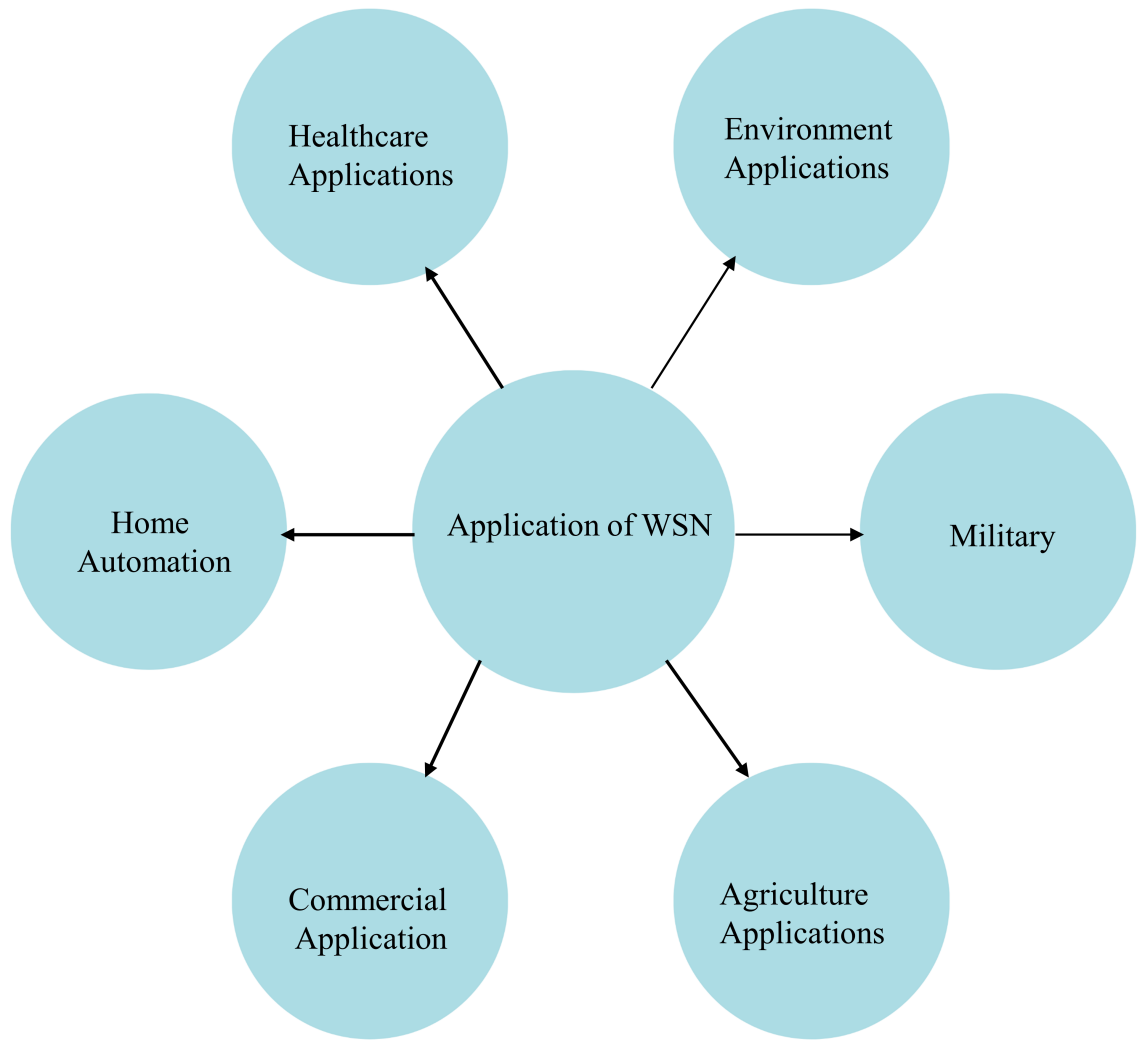
Applications of WSNs across different sectors.

The military, surveillance of healthcare facilities, industrial automation, and intelligent houses are just a few of the applications that employ WSNs. Figure 7 depicts the several uses of WSNs, demonstrating their adaptability [16].

Malevolent actors, however, can quickly inject, intercept, redirect, or modify network connections, revealing vulnerabilities, due to the fundamental features of broadcasting and the sensitivity of wireless networks [35]. This vulnerability raises serious dangers, especially in scenarios where WSNs are critical to healthcare applications, military deployments, or human target recognition. In these cases, a compromise of security can have serious and far-reaching implications. As a result, WSNs have piqued the interest of civilian sectors, notably healthcare. Nonetheless, these networks, which house sensitive data, need strong defences against a variety of possible security risks and assaults. In addition to data security, the continuous flow of information inside these networks is critical and should be preserved.

#### 2.3.1. Military Applications

Improved military command, control, communications, computing, intelligence, surveillance, reconnaissance, and targeting systems are possible thanks to WSNs. They are incredibly well suited for military applications due to their quick deployment, capacity for self-organisation, and durability in the face of errors. Unlike traditional sensors, WSNs, consisting of numerous affordable and expendable sensor nodes, can withstand the loss of specific nodes due to adversarial actions without significantly hindering military activities, rendering them ideal for use on battlefields [36]. Military applications of WSNs span various crucial functions:

- Supervising Allied Personnel, Gear, and Munitions: Military leaders and commanders can oversee the activities of friendly troops, equipment, and ammunition in real time on the battlefield. Small sensors mounted to soldiers, vehicles, and essential supplies continually communicate their status to sink nodes, which send this information to the command. The data can also move up the command structure, where they is combined with data from other units at each level.
- Battlefield Surveillance: Sensor networks cover crucial terrains, approach approaches, and straits quickly and provide attentive observation of opposing forces’ actions. New sensor networks may be quickly established to increase surveillance coverage as operating plans alter.
- Reconnaissance of Opposing Forces and Terrain: Deploying sensor networks in critical terrains allows for the collection of crucial intelligence about opposing troops and the battlefield’s geography in minutes, reducing the chance of enemy interception.
- Targeting: Sensor networks may be linked into intelligent munitions guiding systems to improve precise aiming.
- Battle Damage Assessment: Sensor networks can be carefully put in target locations before or after an assault to capture important data for analysing the level of damage caused.
- Detection and Reconnaissance of Nuclear, Biological, and Chemical (NBC) Attacks: Sensor networks are implemented in friendly regions as warning systems to identify NBC agents in a timely and accurate manner. This prior notice allows for critical reaction time, which reduces casualties. Furthermore, sensor networks may undertake thorough post-attack surveillance, such as nuclear radiations, without exposing reconnaissance crews to potentially dangerous conditions.

#### 2.3.2. Environmental Applications

Sensor networks are critical instruments in a wide range of environmental applications, including wildlife surveillance, planetary exploration, and pollution research. Their ability to collect precise, real-time data contributes considerably to scientific study and sustainable natural resource management. Sensor networks have a wide range of applications in environmental monitoring, contributing to a variety of sectors [37]. These applications encompass the following:

- Forest Fire Detection: Sensor nodes may be carefully placed across woods to create a dense network. In the case of a fire, these nodes can quickly determine the source, allowing for faster response efforts. This approach’s scalability allows for the deployment of millions of nodes connected through radio frequencies or optical networks. Furthermore, to ensure long-term, unattended functioning, these nodes can contain power scavenging technologies like solar cells.
- Biocomplexity Mapping: Mapping environmental biocomplexity needs sophisticated data integration approaches across several temporal and geographical domains. While aerial and satellite sensors are effective for seeing vast amounts of biodiversity, they lack the granularity needed for studying smaller biodiversity aspects. The placement of wireless sensor nodes on the ground bridges this gap, allowing for thorough studies of small-scale biodiversity. These nodes are Internet-connected, allowing for remote monitoring and management of ambient biocomplexity.
- Flood Detection: To identify and monitor floods, systems like ALERT in the United States use a variety of sensors, involving precipitation, water depth, and meteorological sensors. These sensors provide data to a centralised database system, guaranteeing that flood information is prompt and reliable. Decentralised techniques for communicating with sensor nodes in the field are being investigated in research, allowing for both immediate and delayed queries.
- Advanced Farming Techniques: Sensor networks are critical in precision agriculture because they monitor pesticide levels in drinking water, soil erosion rates, and air pollution levels in real time. These qualities improve agricultural practises’ efficiency and sustainability.

#### 2.3.3. Healthcare Applications

Sensor networks have emerged as a transformational force in healthcare, providing a plethora of applications that revolutionise patient care and monitoring. These applications include the following [38,39].

- Assistive Interfaces: Sensor networks play an important role in developing interfaces for people with impairments. These interfaces increase the accessibility and quality of life for impaired people.
- Integrated Patient Monitoring: They are critical components of complete patient monitoring, providing healthcare personnel with real-time access to key patient data such as physiological parameters and vital signs.
- Diagnostics: Sensor networks aid in diagnostic procedures by gathering and analysing data that are critical for illness detection and monitoring. This assists in the discovery and treatment of medical disorders at an early stage.
- Hospital Drug Administration: Sensor networks provide a barrier against drug mistakes in hospital settings. They assist in matching patients with the proper prescriptions by tagging pharmaceuticals with sensor nodes, lowering the likelihood of adverse drug effects.
- Insect and Small Animal Studies: These networks’ scope includes the study of insect and small animal behaviour and physiology. Sensor nodes contribute to scientific studies by allowing for the tracking of these organisms’ motions and internal functions.
- Telemonitoring of Human Physiology: Sensor networks allow for the telemonitoring of human physiological data, which may be retained for extended periods of time and used for medical research. They can also identify and inform healthcare practitioners of major incidents, such as falls among the elderly.
- Tracking and Monitoring Within Hospitals: Sensor nodes linked to patients and medical personnel provide exact tracking and monitoring within hospital grounds. Physicians can quickly identify one another, improving teamwork and efficiency.

The idea of the “Health Smart Home”, which was established in Grenoble, France, to evaluate the practicality of sensor-driven healthcare systems, is an example of modern healthcare breakthroughs. These advancements improve patient care, reduce medical mistakes, and provide patients more mobility and independence. Furthermore, they provide reliable, real-time data to healthcare workers for enhanced decision-making, thus improving the quality of healthcare delivery.

#### 2.3.4. Home Automation

The advancement of technology has led to the emergence of smart homes and intelligent settings, revolutionising the way individuals interact with their surroundings. These developments signify a substantial shift in environmental interaction, offering enhanced comfort, flexibility, and efficiency in both residential and larger areas. The following are some significant developments:

- Home Automation: Smart sensor nodes and actuators may now be integrated into home appliances such as vacuum cleaners, microwave ovens, refrigerators and Video Cassette Recorders (VCRs) thanks to technological advancements. These integrated sensor nodes enable smooth communication inside devices as well as with external networks through the Internet or satellite connections. This interconnection enables users to manage and control their home gadgets effortlessly, whether they are on-site or remotely [40].
- Smart Environments: Human-focused and technology-centric approaches can be employed to approach the idea of intelligent settings. The human-centred approach focuses on adapting the environment to its residents’ individual requirements and preferences, enabling intuitive input and output capabilities. To construct intelligent environments, the technology-centred viewpoint requires the development of cutting-edge hardware technologies, networking solutions, and middleware services [41].

#### 2.3.5. Commercial Applications

The incorporation of sensor networks into commercial and industrial processes has heralded a new era of efficiency, control, and data-driven decision-making across a wide range of industries. Numerous commercial applications make use of sensor network capabilities, revolutionising a variety of sectors [3,42,43,44]:

- Monitoring Material Fatigue: Sensor networks are critical in monitoring material wear and tear and guaranteeing the prompt replacement or repair of fatigue-prone components.
- Virtual Keyboards: Sensor networks are used to build innovative virtual keyboards that provide users with intuitive and responsive input techniques.
- Inventory Management: Sensor-enabled inventory management systems help businesses optimise stock levels and streamline operations by enabling real-time tracking.
- Product Quality Monitoring: By continually monitoring product quality parameters during production operations, sensor networks guarantee stringent quality control.
- Smart Office Spaces: Sensor networks govern ambient variables to increase comfort and efficiency in offices, transforming them into intelligent workspaces.
- Robot Control in Manufacturing: In automated production environments, sensor networks guide and control robots, improving precision and productivity.
- Interactive Toys and Museums: Sensor networks are used by interactive toys and museums to engage people with responsive items, enabling learning and amusement.
- Factory Process Control: Sensor networks are used in industries for real-time process control and automation, guaranteeing efficient and error-free manufacturing.
- Disaster Area Monitoring: In disaster-stricken areas, sensor networks are used to monitor conditions, aid in rescue attempts, and send important data to responders.
- Smart Structures: Sensor nodes are embedded in building infrastructure, allowing for the continuous monitoring of structural health and safety.
- Machine Diagnosis: Sensor networks aid in the detection and diagnosis of equipment problems, hence avoiding costly breakdowns.
- Transportation Solutions: In transportation, sensor networks are used to track and optimise vehicle movements and logistics.
- Vehicle Theft Detection: Sensor nodes are strategically placed throughout a geographic region to detect and identify hazards, instantly reporting instances for study.
- Inventory Control: Sensor nodes may be installed in each warehouse item, allowing for exact tracking and inventory control while minimising mistakes and increasing efficiency.
- Vehicle Tracking: For vehicle tracking, sensor networks provide two options: local determination of vehicle bearing inside clusters and data forwarding to a base station for exact vehicle location determination.

This section has highlighted the numerous uses of WSNs, demonstrating their flexibility to different situations and problems. This flexibility demonstrates the applicability of WSNs in many businesses. Following this examination, the following section will focus on the role of machine learning (ML) in improving WSN performance, with a specific emphasis on data processing and security.

## 3. Machine Learning in WSNs

This section investigates the integration of machine learning (ML) into WSNs, focusing on its rising use in improving essential elements such as data aggregation, anomaly detection, and node positioning. An introduction to the main machine learning techniques used in WSNs will be offered, demonstrating their contributions to improving network efficiency and security.

In the field of WSN security, the use of machine learning techniques is a crucial area of research. We give an overview of several algorithms in this part, categorising them into various groups such as supervised, unsupervised, reinforcement learning, and deep learning. This classification provides a thorough picture, taking into account the numerous machine learning applications within the context of WSN security [45].

Certain WSNs find themselves interacting with very sensitive data in settings loaded with evil intent, frequently unsupervised. Security becomes crucial under these circumstances. This includes addressing data confidentiality, authenticity, integrity, and freshness, all of which are strengthened by stringent security procedures. Because of the inherent constraints in resource availability and the processing capabilities of WSNs, traditional network security techniques such as user authorisation are sometimes impracticable [46].

In another example, a connectivity gateway was developed using the power of ML classification strategies like Random Forest, k-NN (k-Nearest Neighbour), and Naive Bayes in one important research project [47]. When compared to other methodologies, this gateway successfully examines IoT malware network activity, with k-NN displaying excellent accuracy in performance evaluations. Another significant piece of work was a Support Vector Machine (SVM) technique of instruction for Internet of Things (IoT) data. This method significantly decreases computational complexity while avoiding the requirement for a trustworthy third party, indicating a considerable improvement over traditional SVM.

Machine learning technology is emerging as a powerful cost-cutting paradigm in some security sectors. Anomaly detection, for example, has shown amazing results in combating harmful activity in a variety of areas, including packet analysis, protection against Denial-of-Service (DoS) assaults, and network availability enhancement. Along with tackling traffic congestion, error detection and management have embraced the ML technique to improve performance [48]. Furthermore, ML approaches contribute considerably to physical-layer authentication processes, demonstrating their adaptability and accuracy in solving diverse difficulties in WSNs.

### 3.1. Supervised Learning

Supervised learning is a fundamental pillar of machine learning (ML), including the inference of functions from precisely labelled training data sets. These data sets contain training instances, each of which consists of a pair consisting of an input item, generally represented as a vector, and the intended output value, indicated as a trained indicator. As a result, supervised learning algorithms are subjected to a thorough examination of the training data, culminating in the generation of an inferred function. Even when the label is hidden, this function is a powerful tool for mapping and predicting outcomes for new samples. The procedure is dependent on the use of a training set made up of samples with specific properties. A mathematical framework, such as its unique model in recognition of patterns or the weighted model in artificial neural networks, is constructed using this information set and is subsequently used to guide the prediction of unidentified samples [49].

**Figure 8:**
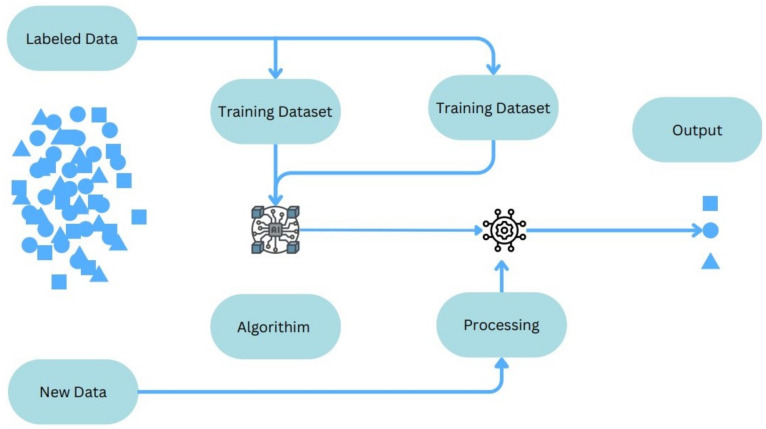
Overview of common supervised learning algorithms in WSNs.

Figure 8 provides a simplified visualisation of the training processes entailed in supervised learning on datasets.

- Linear Regression is a fundamental supervised learning method that predicts a value (Y) based on input data (X). It is a reliable and accurate technique that minimises mistakes, making it a popular machine learning approach. The linear regression model [50] can be represented mathematically as Equation (1). It is useful for addressing various issues, including localisation, connection concerns, data aggregation, and energy harvesting.
  (1) Y=f(x)+ϵ
- Decision Tree (DT): The Decision Tree (DT) is based on the approach of observation-to-value paradigm, in which observations regarding a specific object lead to the assessment of its worth at the tree’s leaves. In the disciplines of statistics, data mining, and machine learning, this technique of predictive modelling is commonly used. Similar to classification trees, decision trees are built using variables that have a set of values and contain leaves that reflect several categories that are represented by branches. Notably, regression trees, a version used for regression analysis, accommodate numeric variables with real values. When dealing with data-driven decision-making, decision trees come to the fore and are useful in data management activities. They are relatively simple to understand in comparison to other classification methods, emphasising their usability [28].
- Support Vector Machine (SVM): The Support Vector Machine (SVM) method is a strong supervised machine learning tool that excels at both classification and regression issues. SVM works by assigning a point in an n-dimensional space to each data piece, with attribute values acting as coordinates. SVM creates a hyperplane in this multidimensional space that successfully divides two unique groups [51].
- Artificial Neural Network (ANN): Data classification using a synthetic neural network (ANN) is a powerful method that is inspired by the complexities of human neuron models. ANNs, which are made up of many neurons or functional units, systematically process data to produce exact results. Levels are widely used in these networks, with linked nodes covering many levels, each with a distinct purpose [52]. An ANN has the structure of three fundamental layers, a layer that provides inputs, one or more hidden layers, and one or more output layers. ANNs excel in classifying complex and non-linear datasets, distinguishing them from traditional classification algorithms due to their absence of input constraints.

### 3.2. Unsupervised Learning

In the extensive field of deep learning and artificial neural networks, unsupervised learning is a critical element. This paradigm makes it possible for machine learning algorithms to find similarities in data without being aware of the data output beforehand, allowing them to handle unlabelled datasets well. Unsupervised learning is commonly used to estimate data density in order to uncover similarities across data points, hence easing statistical organisation. It is worth mentioning that when compared to its supervised equivalent, the fundamental difference is their mode of operation. Unsupervised learning attempts to derive a knowledge-conditioned graphical distribution based on extra parameters [53], whereas supervised learning attempts to infer the inherent distribution of data. Although unsupervised learning has a lower level of complexity and faster implementation, its output prediction accuracy can occasionally fall short. Several important algorithms have emerged in the field of unsupervised learning:

- K-Means Clustering: Uniting Data into Cohesive Clusters The K-means clustering technique is a key component of unsupervised learning. It is an efficient approach for clustering nearby data points into clusters. This method avoids the creation of learning models. Instead, it evaluates each new data point’s closeness to existing clusters, frequently depending on criteria such as distance from the cluster centre or arithmetic mean. The data point is allocated to the bunch to which it is nearest according to these metrics. Consider grouping three distinct clusters from a collection of data points; this entails determining the proximity of each data point to a given cluster by taking criteria such as the Euclidean distance between data points into account [54].
- Fuzzy Logic: Bridging Real Numbers with Degrees of Truth Fuzzy logic is a revolutionary way of introducing degrees between real values. The fuzzy set, under this paradigm, assigns a degree of membership to items inside a universe, commonly stated as a real integer between [0, 1]. This degree of membership is a crucial concept in fuzzy logic that allows statements to have varying degrees of truth. In the classical truth value spectrum, where 0 represents “completely false” and 1 represents “absolutely true”, fuzzy logic provides intermediate values representing partial truth. As a result, fuzzy logic emerges as a potent instrument for dealing with problems marked by imprecision, ambiguity, estimate, qualitative uncertainty, and partial truth [55].
- Hierarchical Clustering: Structured Data Organisation Hierarchical clustering is an unsupervised learning technique that excels in grouping related items into clusters, with top-down and bottom-up approaches available. Top-down hierarchical clustering, sometimes referred to as disagreeable clustering, starts with one major split and recursively divides the data into smaller and smaller clusters until every discovery is given its own cluster. On the other hand, bottom-up hierarchical clustering, sometimes referred to as agglomerative aggregation, assigns each record to a cluster based on a weight per cubic function. Surprisingly, hierarchical clustering is incredibly adaptable and straightforward to use since it does not need previous knowledge of the number of clusters. Data aggregation, synchronisation, mobile sink management, and gathering energy in WSNs are only a few of its many uses [56].

This section looked at how machine learning improves the operation of WSNs, namely in areas like anomaly detection and data optimisation. The major point is that automating decision-making processes improves network performance significantly. The next part will look into various ML applications in WSNs, stressing the approaches’ practical relevance in real-world deployments.

## 4. Applications of ML in WSNs

This section discusses the practical uses of machine learning in Wireless Sensor Networks, with an emphasis on improvements in localisation, security, error detection, and congestion control. The section’s goal is to highlight the actual benefits of incorporating ML into WSNs, particularly in resource-constrained contexts, by looking at real-world instances.

In this part, we will take an in-depth look at the many applications of machine learning (ML) in the complex area of WSNs. Each subsequent paragraph methodically examines the tremendous benefits that result from the prudent adoption of ML approaches to address the diverse difficulties that pervade the WSN ecosystem. Table 3 compares various machine learning algorithms used to address critical challenges in WSNs, such as error detection, security, and overall performance enhancement, highlighting their application areas, assessment criteria, and noteworthy conclusions, providing a more in-depth understanding of how different techniques affect WSNs across multiple parameters.

### 4.1. Localisation

**Figure 9:**
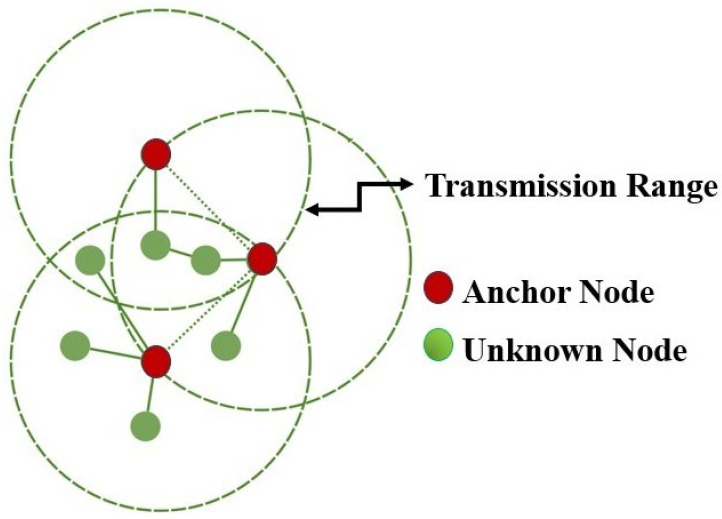
Illustration of localisation techniques in WSNs.

The localisation of sensor nodes in WSNs requires finding their physical or geographical coordinates, a critical issue given the common situation of nodes deployed without prior position knowledge or infrastructure for post-deployment location retrieval. There are several approaches available, such as manual assignment, GPS, or the deployment of specialised nodes such as anchor or beacon nodes. Figure 9 depicts an illustrative case.

Proximity-based, range-based, angle- and distance-based, and known-location-based approaches are among the various localisation modalities [81]. Because of the dynamic of sensor node placements caused by external influences, network reprogramming or reconfiguration is frequently required. In this setting, machine learning (ML) appears as an important technique for improving location accuracy.

ML has a number of advantages. It is excellent at differentiating between network’s anchor nodes and unidentified nodes. ML facilitates the creation of sensor node clusters, allowing for the quick estimation of coordinates for each cluster. In situations where mobile sensor nodes change locations often, ML-based techniques provide a more efficient and accurate method of localisation. Ref. [58] provides a complete overview of ML-based localisation techniques in WSNs. In one prominent paper, scientists presented a method for improving localisation accuracy by combining k-means and fuzzy c-means algorithms. To train the system, these clustering algorithms were only used at the washbasin. Depending on the Received Signal Strength Indicator (RSSI) values, the area was divided into clusters, and each cluster was separately trained to determine the locations of the sensor nodes.

Another technique [59] used a unique PSO-based Neural Network (LPSONN) scheme to handle localisation in WSNs. Anchor nodes computed their hop counts to other anchor nodes in this system and sent this information to a head anchor node. LPSONN focuses only on the neural network training of the head anchor nodes, with a PSO method applied to optimise hidden layer quantities. When compared to current approaches, this strategy outperformed them, resulting in reduced error rates. An energy-efficient distance estimate approach based on artificial neural networks (ANNs) was presented to fight difficulties such as [60] anisotropic signal attenuation interference in shadow and fading circumstances. This ANN-based approach demonstrated robustness in a variety of metrics, including accuracy.

In another work, the authors [57] introduced a mobile sensor node localisation strategy that uses ANN and improves performance using Particle Swarm Optimisation (PSO). In comparison to conventional methodologies, SO was utilised to calculate the right number of neurons required for efficient localisation in dynamic WSNs. An technique combining fuzzy logic and vector PSO for range-free localisation was developed in circumstances including non-uniform node placement. When compared to previous localisation algorithms, this strategy prioritised diverse circumstances, resulting in less complexity.

Another technique [17] used fuzzy language modelling to focus on indoor localisation for mobile sensor nodes. This approach handled RSSI variances using interval type 2 fuzzification and fuzzy spatial marker (FSM) identification for geometric re-division detection. A graph-based localisation system combining Support Vector Machines (SVMs) and Conventional Neural Networks (CNNs) was presented in another article. This strategy [82] improved classification accuracy by analysing target signals and signal intensity using heterogeneous dual classifiers. It also pinpointed leakage points, decreasing mistakes and correcting time synchronisation difficulties.

In order to localise large WSNs, Support Vector Machines (SVMs) were used. To decrease training costs and learning calculations, the sample technique was modified using fuzzy c-means [83]. This procedure not only increased localisation accuracy but also addressed difficulties with coverage gaps and border areas. Another work [17] described a range-free localisation algorithm based on SVM that used a transmit matrix to construct correlations between hops and distances. To detect unknown nodes inside the WSNs, SVM was utilised.

Another technique [84] divided the WSN area into discrete grids using SVM classification and a polar coordinate system. When paired with a two-hop mass-spring optimisation (THMSO) strategy, this method resulted in improved localisation efficiency. Using iterative variations [26], in order to eliminate off-grid mistakes and recover faulty prior information, Bayesian procedures for estimating and customisation employed accurate sparse replacement techniques and a three-level structure prototype.

To handle outliers, a non-convex robust principal component analysis (PCA) method was developed, with the goal of reducing dimensionality for more efficient and accurate results. For sensor node localisation, weighted linear regression was used, with neighbouring anchor nodes receiving more weight. This method outperforms several anisotropic network topologies. Based on the Swarm Intelligence Optimisation method, an optimum relay node distribution algorithm optimised relay node placements, minimising energy usage while sending information from source to base station [85]. Localisation via a semi-supervised learning solution for mobile WSNs permitted the integration of new sensors and adaption to environmental changes without needing algorithm adjustments. Even with insufficient labelled data, the proposed method achieved significant accuracy.

A dynamic localisation strategy for mobile sensor nodes was presented in the research [86], which takes into account both interior and outdoor situations. This method’s temporal complexity increases with the number of nodes and is based on semi-supervised Hidden Markov Models (HMMs). For mobile WSNs, a semi-supervised learning technique was suggested that reliably determines sensor node positions utilising primarily unlabelled data and minimum labelled data received from signal and physical space. Support vector regression was used for target localisation in a [87] combination of semi-supervised learning and support vector regression after the system first completed training with a small sample of labelled data. This method produced exact findings and minimised memory utilisation by using a simplified support vector regression methodology.

### 4.2. Anomaly Detection

Anomalies in WSNs, as evidenced by inconsistencies in data readings, are a major source of worry [62]. These anomalies might be caused by inconsistencies in sensor data measurement or interruptions in traffic-related parameters. Sensors continuously gather environmental data in many WSN applications and send them to a base station through intermediate nodes. The danger of data loss owing to anomalous assaults during data transmission needs the security of sensing data. In WSNs, anomaly detection is critical for minimising communication overhead while transferring information across sensor nodes.

**Figure 10:**
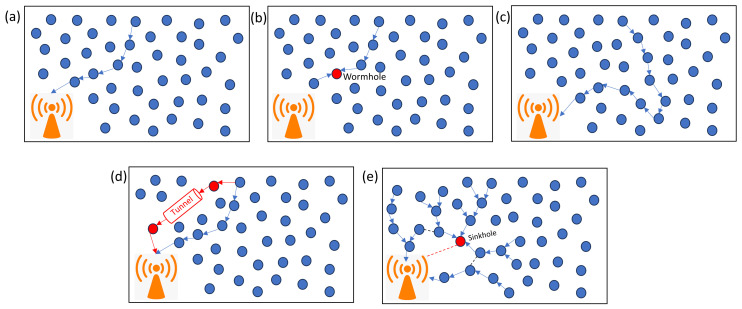
Common anomaly detection methods for WSN security: (**a**) normal flow, (**b**) black hole attack, (**c**) misdirection attack, (**d**) wormhole attack, (**e**) sinkhole attack.

Blackhole attacks, misdirection attacks, wormhole attacks, sinkhole attacks, and hybrid anomalies are all possible threats to WSNs [62]. Figure 10a depicts the overall data flow in WSNs. A hostile node intercepts packets instead of passing them to the base station in a blackhole attack (Figure 10b). Misdirection attacks (Figure 10c) include attackers directing packets through nodes that are not their immediate neighbours, which can result in longer paths and lower performance. Wormhole attacks (Figure 10d) establish tunnels between distant nodes, giving the appearance of near proximity and allowing attackers to redirect or collect packets. Sinkhole attacks (Figure 10e) include a specific node advertising an ideal path to neighbouring nodes, altering data, and causing network disruption. Hybrid assaults mix several attack kinds, adding to the difficulty. Machine learning (ML) techniques are used to identify abnormalities in WSNs, protecting them from a variety of assaults and irregularities.

Multiple algorithms, such as autonomous threshold learning and slumber coordination methods for diverse sensor networks featuring geological sensor nodes, have been used to protect WSNs from anomalies, as well as for detecting sinkhole attacks and denial-of-service (DoS) attacks by identifying inconsistent data. Ref. [88] presents an overview of anomaly identification in non-stationary datasets using ML. ML-based anomaly detection in WSNs has various advantages, including the ability to handle hybrid abnormalities quickly, minimise complexity and communication cost via clustering techniques, and dynamically change parameters based on previous data.

The research [62] proposes a method for identifying hybrid assaults using the k-means clustering approach. During blackhole and misdirection attacks, this technique effectively identifies hostile nodes. A hypergrid k-NN algorithm is utilised in [61] for online anomaly detection, specifically to protect against random errors and cyber-attacks. The use of hypergrid instead of k-NN improves anomaly detection while satisfying certain WSNs constraints. Hypersphere detection zones are renamed hypercube detection regions for online anomaly detection to reduce computational and communication overhead.

An intrusion detection system (IDS) design adopts a Decision Tree classification technique in this work [89], primarily for environmental monitoring. The authors [90] use two ML-based techniques to detect outliers in WSNs: SVM with sliding window and Principal Component Analysis using the method of least squares. To improve anomaly identification in non-stationary time-series data, a modified RBF kernel and PCA are used. Utilising the Support Vector Data Description (SVDD) classifier [91], abnormalities in node data are found while simultaneously reducing the complexity of both the training and testing phases. To decrease training phase complexity, a two-order approximation-based Sequential Minimal Optimisation (SMO) approach is utilised, while a quick decision-making technique in the testing phase depends on the hypersphere’s centre point in the kernel feature space. The authors [92] provide a comprehensive review of several outlier discovery strategies utilising one-class SVM in demanding contexts, highlighting the computational efficiency and communication overhead benefits of the quarter-sphere formulation for outlier detection.

### 4.3. Error Detection

Machine learning techniques are extremely useful in error detection, particularly in the setting of WSNs, which are susceptible to a range of challenges related to software, hardware, and implementation in various domains. The rapid discovery of defects in WSNs demands the use of strong error detection algorithms. The authors of one study used a trust mechanism decision fusion approach, boosting its performance using four categorisation strategies: K-Nearest Neighbour (k-NN), Extreme Learning Machine, Support Vector Machine (SVM), and Recurrent Learning Machine. However, this technique does not capture the dynamics of individual WSNs node failures [63].

Another technique uses a Hidden Markov Model (HMM) to comprehend the dynamics of transitions caused by errors to alleviate this issue and dynamically capture WSN node malfunctions during fault occurrences. By relying on state transition probabilities produced by the Markov model, neural networks successfully classify mistakes. This approach combines a Hidden Markov Model with neural network techniques including Learning Vector Quantisation, Probabilistic Neural Network, Probabilistic Adaptive Neural Network, and Radial Basis Function [64].

Support Vector Machine (SVM) classification is used in the field of traditional machine learning (ML) techniques for error detection in WSNs. In a similar spirit, SVM regression models are used in another work [93] for the same objective. In addition, a technique for detecting errors in data flows in real time employs Recursive Principal Component Analysis (PCA) and a multi-class Support Vector Data Description (SVDD) classifier. The SVDD classifier classifies error types, while the lightweight recursive PCA approach identifies faults in WSNs [65].

**Figure 11:**
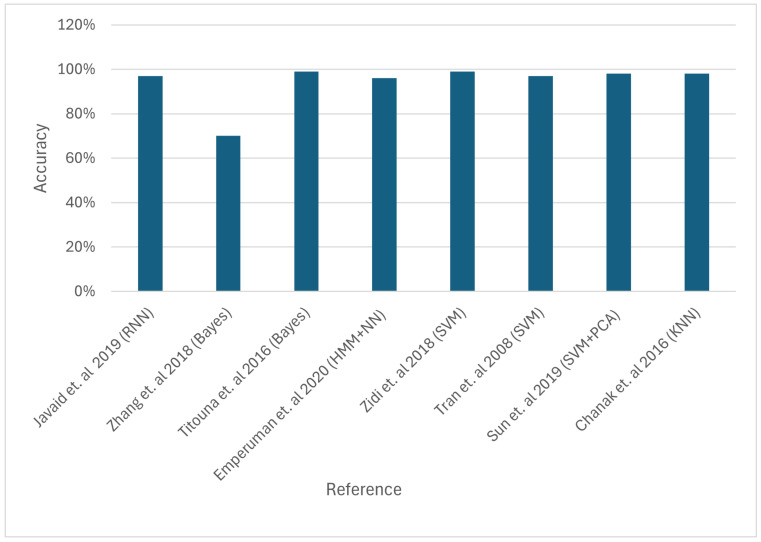
Comparison of error detection techniques in WSNs [63,64,65,93,94,95,96,97].

Error detection is critical in body sensor networks because failing to recognise a malfunction might lead to incorrect medical diagnosis. A Bayesian network-based error detection technique that captures temporal and spatial connections among body sensors is suggested. Setting proper thresholds allows this approach to determine sensor faults [94]. In addition, an error detection method for locating problematic nodes in WSNs is provided. This method is based on battery and WSN node data. In order to find errors within sensor nodes, a Naive Bayesian classifier is utilised in the first stage. The second stage evaluates fault detection via the block header or gate. Figure 11 compares the accuracy of different approaches; however, it is important to note that the simulation conditions, including network size, energy availability, and data load, vary significantly across the studies. To ensure a more accurate comparison, future research should standardise these variables or, at the very least, present the results as normalised data relative to baseline conditions. The simulation results show almost 100% accuracy rate [95].

In wireless networks, another technique based on fuzzy criteria presents a flawed node allocation and management strategy. The main goal is to repurpose failing WSN nodes by providing optimal paths to the base station, hence improving service dependability and network lifetime. A K-Nearest Neighbour (k-NN) classifier is used to detect anomalies in sensor behaviour, depending on the error rate of WSN nodes, to discover malfunctioning nodes [96].

### 4.4. Target Tracking

The identification and monitoring of particular stationary or mobile entities inside WSNs comprises target tracking. Target tracking in WSNs can be accomplished with a single sensor node or many nodes, depending on the application’s needs. Using a single node for tracking reduces energy usage; however, numerous nodes contribute to more exact findings. The pursuit of high-quality target tracking, on the other hand, offers various obstacles, including node failures, missing targets, coverage and connection issues, data aggregation complications, tracking delay, and energy consumption concerns. Ref. [98] provides a thorough examination of target tracking systems.

To solve these issues in target tracking within WSNs, many techniques have been proposed. Another line of investigation is the estimation-based target tracking technique described in [66]. Machine learning (ML) methods have garnered increasing attention as a means to enhance the precision of target tracking in WSNs. Here are a few of these strategies:

- Computational Overhead Reduction: ML approaches, whether used with fixed or mobile sensor nodes, reduce the compute needs for monitoring mobile objects.
- Adaptability to Dynamic Targets: Given the changing nature of targets in sensor networks, ML techniques optimise target tracking efficiency.

The authors [99] suggest a target tracking approach for WSNs, as well as data fusion using a Bayesian posterior. The accuracy of the technique is demonstrated using both computer-generated data and real-time situations. Within WSNs, this method produces precise target tracking and data fusion. A multi-layer dynamic Bayesian network (MDBN) is introduced in this paper [67] for mobile target tracking. Even in circumstances with nonlinear data and time-varying Received Signal Strength (RSS) precision, Bayesian statistics considerably improve target tracking accuracy. When compared to traditional approaches, this method offers a promising approach to optimum target tracking.

The authors [68] offer a Bayesian learning-based node selection technique for signal recovery. This technique effectively opts for a subset of sensors for target selection, decreasing communication overhead and allowing for fast signal recovery. Researchers [100] use a Bayesian filtering approach for multi-target tracking in WSNs. Through Bayesian approaches, this two-layer system handles information fusion and target tracking. The computational overhead is reduced and energy consumption across sensor nodes is balanced by using this dual-layer topology. In the study [101], two machine learning methods, Bayesian and reinforcement learning, are applied for event monitoring. A dynamic Bayesian network watches the event, while reinforcement learning optimises sensor node sleep cycles. In terms of energy usage and data accuracy, this technique is quite effective.

The proposed [102] WSNs uses chemocapacitor-based sensor arrays and powerful machine learning methods like support vector machines and random forests to monitor environmental variables in real time. Experiments on identifying volatile organic molecules revealed good selectivity, sensitivity, and long-term stability. Machine learning increased sensor efficiency by reducing human interaction, but its limits include complexity in large-scale implementation and reliance on robust data pretreatment. While the system functioned effectively in controlled settings, additional standardisation and adaptability to different surroundings are required for general use.

The task scheduling method in [103], which is based on Q-learning and a shared value function, enables sensor nodes in WSNs to dynamically adapt to environmental changes. The technique minimises the frequency of cooperative information sharing while remaining efficient. Experimental findings demonstrated enhanced application performance and energy usage as compared to traditional approaches. However, despite improved performance, QS has problems in dealing with high energy consumption during cooperative learning. Its merits are in efficiently balancing exploration and exploitation, but real-world implementation remains challenging.

The study [104] proposes the Hybrid Memetic Framework for Coverage Optimisation (Hy-MFCO), which combines a memetic algorithm (MA) with a heuristic recursive algorithm (HRA) to maximise WSNs coverage. To conserve energy, the MA divides sensor nodes into separate groups, whereas the HRA dynamically adjusts for coverage shortfalls by activating hibernating nodes. The experimental findings reveal that Hy-MFCO beats previous approaches in terms of energy efficiency and coverage preservation. However, its intricacy may cause higher calculation time compared to simpler heuristics.

The STLGBM-DDS method combines LightGBM with data balancing techniques such as SMOTE and Tomek-Links to identify DoS attacks in WSNs. The suggested [105] approach improves efficiency by combining feature selection with the Information Gain Ratio. The experimental findings reveal that STLGBM-DDS outperforms standard and hybrid machine learning algorithms, obtaining 99.95% accuracy. While the strategy is excellent at dealing with uneven data, it increases computing complexity. High precision, good data imbalance management, and better feature selection are among the strengths. The limitations include vulnerability to overfitting and increasing computing needs.

### 4.5. Authentication

**Figure 12:**
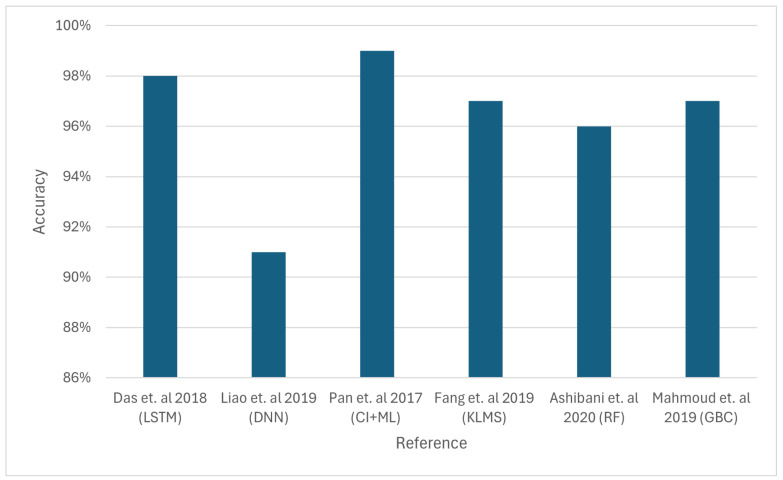
Performance comparison of machine learning models for authentication in WSNs [69,70,71,107,108,109].

Authentication, an important aspect of security in WSNs, ensures the integrity and origin of data while preventing active threats such as DoS attacks and spoofing [106]. It has two dimensions: network components and message properties. Entity authentication validates the identification of the entities involved by allowing the claimant and verifier to interact while exposing minimal information. Message authentication, on the other hand, does not specify the date of message production but does ensure appropriateness. Authentication in traditional networks is based on standard public-key cryptography methods and algorithms such as RSA, ECC, Defihelman, and others. However, implementing these processes within the confines of WSNs frequently results in power depletion. To overcome this issue, utilising the physical layer for authentication appears to be an appealing alternative, particularly in contexts where high processing and battery capabilities are absent. Figure 12 compares authentication systems using machine learning, but future research should standardise simulation conditions or present normalised data to ensure accurate comparisons.

Through physical-layer authentication, machine learning approaches have showed promise in lowering power usage in WSNs. The physical-layer authentication system introduced by [69] relies on Long Short-Term Memory (LSTM) as its cornerstone. This method distinguishes low-power transmitters from high-power ones by analyzing the temporal correlation between the I (preamble phase) and Q (quadrature phase) components of wireless signals. Deep learning techniques, such as LSTM, significantly outperform classical ML algorithms in terms of accuracy.

The deep-learning-based physical-layer authentication framework in [70] uses three algorithms—DNN-based, CNN-based, and CPNN-based—to improve security in industrial WSNs. DNN-based approaches are superior in performance but need significant resources, making them suited for smaller CSIs. CNN-based algorithms are efficient, but they may lose data during convolution. The unique CPNN-based technique balances performance and training time by preprocessing data, making it ideal for real-time applications. Experiments demonstrate high authentication rates (up to 99.5% with DNN) but highlight that CNN has longer training times compared to CPNN.

The authors [107] employ radio channel information and machine learning methods to authenticate WSNs nodes. This approach identifies unauthorised access by comparing radio channel similarities between legitimate and illegitimate users at predetermined intervals. The algorithm learns from previous timeslot experiences and adjusts to the system state, resulting in a success authentication rate of over 99.8% over 1000 timeslots, beating fixed threshold techniques in dynamic contexts. The experimental findings, which were simulated using the USRP platform and MATLAB, indicate the method’s effectiveness in determining the ideal threshold. The algorithm’s strength is its flexibility and capacity to learn from experience, but its weakness is its reliance on a tiny probability to keep the searching algorithm from becoming locked in a local optimal trap.

The study [108] presents a physical-layer authentication approach based on channel information and machine learning with a dynamic threshold, employing a c-greedy strategy to calculate the best threshold, and attaining a success authentication rate of more than 99.8% in 1000 timeslots. This methodology beats fixed threshold approaches in dynamic contexts, demonstrating its versatility and capacity to learn from experience. However, its dependence on a low likelihood of avoiding local optimal traps is a weakness. The suggested technique outperforms previous methods, such as those that use the Q-learning strategy, but may require more CPU resources owing to its machine learning component. Overall, the algorithm’s capacity to adapt to changing system conditions and learn from previous experiences makes it a reliable solution for physical-layer authentication in dynamic wireless contexts.

The study [71] describes another novel solution based on authentication interval logs. It employs feature selection and machine learning (ML) algorithms (e.g., K-Nearest Neighbours, Random Forest, Multi-Layer Perceptron, and Gradient Boosting) on access logs. This method identifies authentication policies while also proving authentication accuracy and showcasing the usage of protocol access history to build user authentication models. However, it is crucial to remember that these strategies are most successful when WSN nodes change locations on a regular basis.

### 4.6. Congestion Control

**Figure 13:**
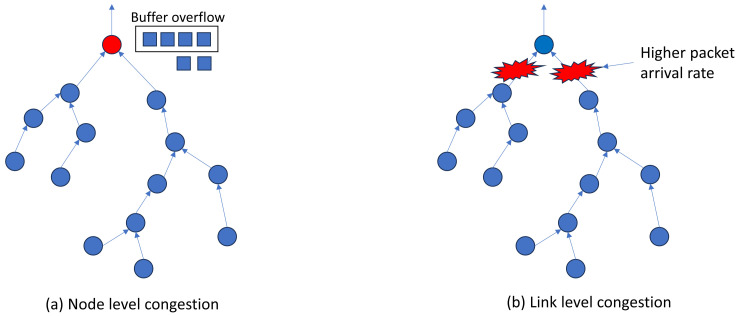
Illustration of congestion scenarios in WSNs.

When a sensor node or communication link is subjected to a volume of information transfer that is greater than its capacity, congestion in WSNs occurs. Node buffer overflow, one-to-many data transmission methods, competition for transmission channels, packet collisions, varying periodic patterns, and transmission rates are some of the variables that cause congestion [110]. For an illustration of congestion across the node and link levels, see Figure 13. Figure 13a shows the node-level congestion induced by a high packet arrival rate, whereas Figure 13b shows the link-level congestion brought on by collisions and reduced bit transmission rates between two nodes.

Numerous crucial WSN indicators, including energy use, Quality of Service (QoS), end-to-end delays, and packet delivery ratios (PDRs), are impacted by congestion. Congestion management is therefore one of the most important problems in WSNs. Congestion control algorithms [111] are the outcome of recent research and are aimed at enhancing energy-conscious data routing. Although they have the following advantages, machine learning (ML) technologies have greater potential for reducing congestion.

- Enhanced Traffic Estimation: Machine learning approaches excel at assessing traffic loads and establishing effective pathways to minimise end-to-end delays between nodes and the base station.
- Dynamic Transmission Range: ML enables the dynamic modification of transmission ranges in response to changing network dynamics.

Congestion has direct consequences for packet loss, energy consumption, and end-to-end delays. In response to these problems, the publication [74] proposes a neural-network-based data compression strategy to decrease congestion inside WSNs while also balancing sensor node energy consumption. This method is based on sending compressed data between nodes or Cluster Heads (CHs), which efficiently minimises communication overhead, resulting in lower energy consumption and increased resistance to congestion. This technique, in particular, beats several existing approaches in handling both spatial and temporal data compressions, making it a powerful tool in congestion control.

This paper introduces a congestion diagnosis and management technique using fuzzy logic [72] to lower packet loss rates through the use of efficient and proactive queue control. The strategy is divided into three key stages. It first marshals the queue, using a fuzzy approach to detect congestion. Following that, it orchestrates congestion control, ultimately resulting in congestion recovery by harmonising data flow.

The author of [75] presents a WSN-specific SVM-based congestion management technique. This strategy requires dynamically adjusting transmission rates for each node to accommodate changes in traffic patterns. When compared to other classification algorithms, SVM comes out on top in terms of precision.

The paper [112] presents a multi-agent reinforcement learning technique for a more energy-efficient data collection strategy within WSNs. This method implies traffic management at the cluster-ring level, making optimum routing decisions. It also modifies routing patterns to meet dynamic changes in network topologies, resulting in more effective congestion management.

### 4.7. Diversified Security

Scientists used neural network methods in their study [113], utilising three primary neurons representing devices, sensing, and latency, enhanced by five hidden neurons scattered over three layers. This programme examined the life of individual nodes by analysing the properties of network packets. Any variations from predicted values were used to identify possible problems, such as disinformation or potential man-in-the-middle attacks.

**Figure 14:**
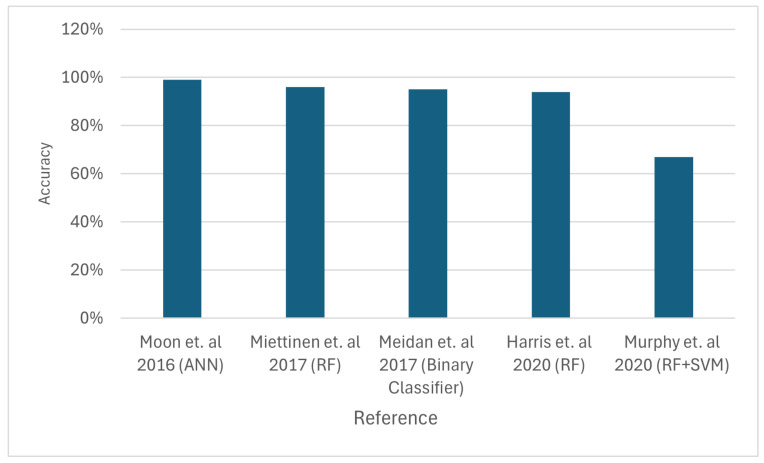
Diversified security performance comparison [73,112,114,115,116].

Another unique method based on machine learning techniques was presented in [114], where a novel model was established to classify newly connected devices in home or office settings into two trustworthiness categories: strict or limited. A gatekeeper was in charge of traffic emanating from these newly integrated devices. It created one-of-a-kind device fingerprints, which were subsequently sent to an IoT security service provider. A comparison of the performance of various security systems utilising machine learning is presented in Figure 14, although it is essential to standardise simulation conditions or normalise data in future research to facilitate accurate comparisons. Based on the device’s kind and traffic patterns, this supplier used machine learning classification algorithms to infer its nature.

The authors [115] used machine learning to differentiate between WSNs and non-WSN devices, depending mostly on data traffic for categorisation. The classification technique relied on sessions and derived information from the behaviour of each device. An innovative method described in [117] sought to detect the change of a benign node into a malignant one using machine learning techniques. This method used bio-inspired concepts similar to those found in the immune system. Data were initially divided into two distinct groups by the k-means algorithm: normal and flawed. Then, a decision framework with three primary regions—normal, fault, and critical borders—was constructed using Support Vector Machines (SVM). The SVM dataset aided in the estimation of the WSN node’s mean and standard deviation. Anomalies discovered using this method prompted the deployment of a virtual immune system. This technology generated virtual antibodies, which efficiently neutralised hostile nodes and mimicked biological immune responses.

In the context of WSN privacy and security, researchers [116] examined the applicability of machine learning algorithms for interference recognition. They mainly studied samples in the in-phase (I) and quadrature (Q) phases. The need for interference awareness was emphasised by the mitigation measures that were put in place after intrusions were discovered. Researchers used the Random Forest classifier because to its aggregation of several independent decision trees working together. The performance of Random Forest and SVM classifiers in WSN channel identification was then examined by the same authors in [73]. They gathered data on the properties of the I and Q samples, and they added data from wired devices that were not hampered to this dataset. This rich dataset was trained using machine learning methods before being evaluated in situations involving signal-free circumstances, genuine signals, and jamming signals. The outputs of these classifiers were meticulously compared against data acquired from interference-free wired sources.

### 4.8. QoS

The degree of service quality (QoS) provided by WSNs has a considerable impact on user happiness and system effectiveness. The network-specific and application-specific aspects of QoS in WSNs may be divided into two categories. The former includes crucial characteristics such as sensor node energy consumption rate and bandwidth availability, whilst the latter includes the network’s operational parameters, including sensor node measurements, their positioning, and the quantity of active nodes in the network, are assessed. Stringent resource limits, unequal traffic distribution, data redundancy, network dynamism, energy equilibrium, scalability issues, the presence of many sinks, and diverse traffic kinds are all problems for QoS preservation in WSNs [118].

A comprehensive overview of machine learning (ML)-based approaches dedicated to enhancing QoS within WSNs is thoughtfully presented in Table 4.

In a groundbreaking effort described in [119], researchers offered an energy-efficient approach to data fusion based on fuzzy logic, a technique aimed at attaining improved QoS within WSNs. Unlike traditional approaches, which frequently collect entire datasets from WSNs, this algorithm selectively consolidates only verified information. Specifically designed for cluster-based sensor networks, each Cluster Head (CH) is tasked with acquiring information from its member nodes in response to the detection of a certain event. The deployment of fuzzy logic controllers integrated into each sensor node is the beauty of this strategy. Before relaying information to the nearest CHs, these controllers thoroughly verify its accuracy.

While this is happening, a ground-breaking answer to the problem of how WSNs and smart energy systems interact, known as the Wavelet Neural-Network-based Line Quality Estimation (WNN-LQE) procedure, emerges in [76] this paper. Its major objective is to improve the Quality of Service (QoS) inside these complex networks. This novel technique assesses multiple connection quality characteristics using the signal-to-noise ratio (SNR) concept. Furthermore, it expertly reduces communication overhead while orchestrating a fair distribution of energy resources, eventually improving network QoS.

A cross-layer Communication Protocol, as disclosed in [77], employs a multi-agent reinforcement learning framework in the persistent quest for efficient self-organisation inside WSNs. This clever technique is a critical building piece in developing an adaptable and harmonious WSN ecology. Similarly, ref. [78] presents a complex system that combines multi-agent reinforcement learning, energy-aware topology control, and data dissemination protocols. This comprehensive technique enables the network to deliberately pick active neighbour nodes, ensuring the network’s strong structure. It also uses a convex-hull technique to maintain connection and coverage for border nodes. The net result is an optimised QoS, which considerably improves the performance of the sensor network.

Authors [120] demonstrates a Q-leaning-based technique precisely constructed to address the dual difficulties of QoS uncertainty and dynamic service behaviour in the arena of QoS and WSNs. This advanced system shines as a light of hope, establishing global excellence in safety services. Ref. [121] proposes Distributed Adaptive Cooperative Routing Protocols (DACRs), a sophisticated system powered by a lightweight reinforcement learning algorithm. The operation of DACR focuses on the prudent selection of optimum relay nodes and intelligent decision-making about transmission modes at each node. The primary objective is to maximise network stability, eventually providing WSNs with stable and dependable QoS.

This section has uncovered the many uses of machine learnng in WSNs, highlighting how these approaches improve network performance and security. One important conclusion is that ML may be adjusted to meet specific WSNs requirements, such as enhancing localisation accuracy and identifying data abnormalities. Building on these practical applications, the following section will investigate the use of machine learning and Blockchain for enhanced cyber-attack protection in WSNs.

## 5. Cyber Attacks Prevention Using Machine Learning (ML) and Blockchain (BC) in WSN

This section investigates the growing demand for reliable security in WSNs, with an emphasis on the combined use of machine learning and Blockchain technologies to identify and prevent cyber-attacks. The discussion focuses on the advantages of combining ML with Blockchain, stressing their ability to strengthen security protocols, maintain data integrity, and increase system resilience.

### 5.1. Machine Learning (ML) and Cyberattacks

Machine learning (ML) algorithms are used to build autonomous classifiers based on behaviours by utilising mathematical methodologies based on specialised datasets. There is no need for human interaction because these classifiers can work autonomously. These techniques improve the network nodes’ capacity for implicit programming-free autonomous learning and employ these models to provide predictions based on fresh input data. Smart cities, energy management, agriculture, intelligent transportation systems, industrial and manufacturing processes, search engines, social media, cyberattack detection, spam email filtering, and recommendation systems are just a few of the domains where machine learning (ML) techniques are used. Through the use of various ML techniques, they, for example, improve operations like data sensing, cluster head (CH) selection, routing path determination, data aggregation, reducing packet delivery latency, duty cycle optimisation, Quality of Service (QoS) provisioning, resource management, and extending network lifetime.

In order to protect WSNs from cyberattacks, lightweight detection and mitigation systems have also been made possible using ML algorithms. These algorithms provide sensor nodes with the ability to identify possible dangers, raise an alert, evaluate the risk, and isolate the offending node, therefore reducing the effect on the network’s development. The data collection, preprocessing, feature selection, model training using appropriate ML techniques, hyperparameter tweaking, model testing, validation, and deployment operations are all included in the ML pipeline.

### 5.2. BC Introduction

The researchers in [122] first presented digital timestamping, an early data security technique, in 1991, and it has since attracted a lot of attention from the academic and professional worlds. Their main suggestion was to use a variety of hash functions to avoid collisions, digital signatures, and connecting techniques to maintain the network’s client requests’ sequential sequence. Digital timestamping is a forerunner of the well-known Blockchain (BC) technology [123], which will be discussed in the parts that follow. According to block and transaction sizes, BC is made up of blocks, each of which aggregates a certain collection of transactions. These blocks are connected using cryptographic sequential digital signatures that use a hash value obtained from both previous and current blocks to protect the content within the blocks from unauthorised changes. This sequence begins with a fundamental block that is designated as the series’ first block and is known as the genesis block [124]. Through a consensus procedure including a timestamp and hash, each succeeding block is added. A consensus method updates each node linked to the Blockchain (BC) network’s shared ledger upon the addition of a block, creating a consolidated ledger that is immutable.

Blockchain (BC) eliminates the need for a centralised third-party organisation by operating in a distributed or decentralised manner and supporting all network transactions, making it essential for protecting cryptocurrency networks. Additionally, BC has a structure that is ideal for a variety of applications that call for distributed transactions between nodes and decentralised computing and administration in a trustless environment [125]. By enabling safe data storage, routing, resource access, and identity authentication, it can be incorporated into IoT systems to reduce security concerns [126]. According to [107], because to its scalability and quick settlement capabilities for coordinating and protecting joining nodes, BC’s peer-to-peer (P2P) distributed ledger makes it a good choice for securing IoT data and confirming identities. However, the difficulty in deploying BC in WSNs is the increased demand for storage and processing resources, which results in increased latency and decreased network throughput. Contrary to sensor devices, which are often designed to be resource-restricted and cost-effective, BC frequently involves increased communication costs, memory usage, and energy consumption. However, by integrating BC, the cost associated with creating and maintaining a centralised database may be reduced. Utilising all of a node’s computational, storage, and bandwidth capabilities, it takes advantage of a node’s idle status to reduce the cost of network processing and storage.

### 5.3. Securing WSN

A typical Blockchain (BC) operation in a peer-to-peer WSNs starts with a transaction between two sensor nodes. This transaction is distributed around the P2P network after being hashed. Each transaction in a multi-path forwarding situation is individually signed using the public keys of the participating nodes. Through the consensus method, miners or voters examine and verify transactions involving data and identity. These verified transactions are then distributed, recorded, and collected into a block. As soon as the block is finished, it is added to the BC P2P network, strengthening the shared, unchangeable, and impenetrable ledger between member nodes.

**Figure 15:**
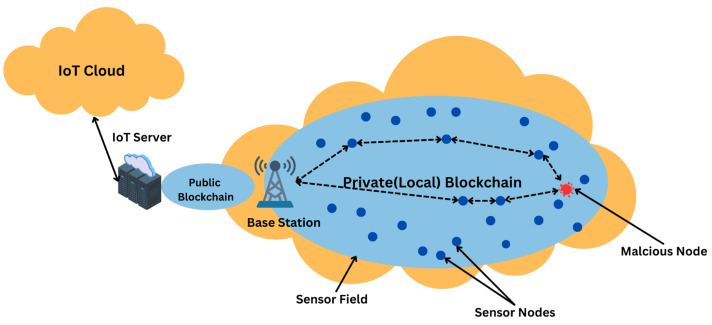
Centralised design for securing WSNs using Blockchain.

**Figure 16:**
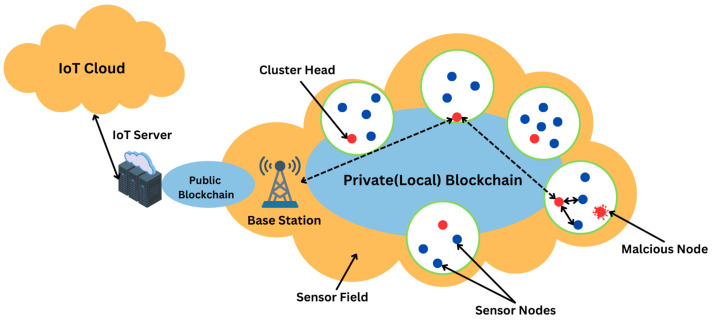
Cluster-based design for WSN security using Blockchain.

Two common architectures for BC-based security systems exist in the context of WSNs: centralised (Figure 15) and cluster-based (Figure 16). Two node kinds are also present: full and lightweight. Full or aggregator nodes have access to the complete chain’s transaction history because they locally store the entire ledger [127]. Unlike lightweight nodes, which selectively keep BC transactions relevant to their operations, these nodes, which are frequently base stations (BS) or cluster heads (CH), do not. Lightweight nodes [128], usually terminal nodes or cluster members (CM), rely on connected full nodes, which minimises the number of data they must download and store. The deployment of a single central node as a master authority, analogous to a BC or CH, responsible for authentication and trust management, is prohibited despite the fact that these architectures are compatible with private BCs in order to reduce important points of vulnerability within the network.

**Figure 17:**
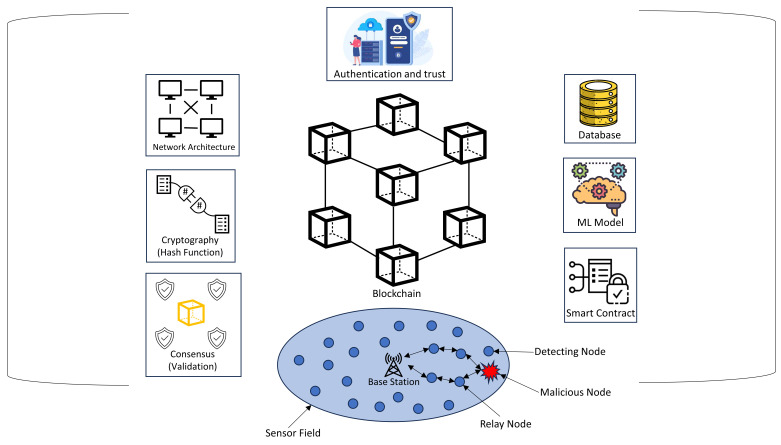
Important features of Blockchain–machine-learning integration for securing WSNs.

A viable strategy for improving WSN security is the combination of Blockchain (BC) and machine learning (ML). ML provides reliable attack identification, while BC assures safe and tamper-proof data recording. This integration makes use of BC to safely store the enormous amounts of data that WSN devices create. The data may then be arranged for ML training, potentially leading to high detection accuracy. To keep detection integrity, the ML output can be safely kept on the BC network. Combining these technologies creates new difficulties but also has significant advantages. Key components of the combined BC-ML security method are shown in Figure 17.

BC is used for attack prevention in this strategy [129] while ML is used for attack detection. When the first line fails, the second line scans the incoming traffic for holes and warns the network of potential threats. A relatively recent research area with tremendous potential is the combination of BC and ML for WSN security.

This has been investigated to secure WSN routing protocols by integrating BC-ML. In order to reduce packet delay and transaction latency, BC securely stores routing information and upholds data integrity. Techniques for detecting malicious nodes utilising BC-ML integration have also been studied. High detection performance is shown by anomaly detection models integrating BC and ML [79] with less communication and storage overhead. ML-based identity management and secure routing models have demonstrated promise in identifying rogue nodes and boosting WSN security. This line of inquiry has enormous promise for enhancing WSN security in the face of developing online dangers.

Future research should [130] prioritise the creation of a solid integrated solution fusing Blockchain (BC) with machine learning (ML) to improve the security of WSNs. To carry this out, a lightweight framework that effectively secures WSNs in untrusted contexts must be created while taking into account the resource constraints of sensors. It is essential to develop specialised consensus protocols, smart contracts tailored for particular applications, accelerated transaction validation, alternate block generation techniques, and optimised designs that balance computation and communication among nodes in WSN deployments in order to accomplish this goal.

**Figure 18:**
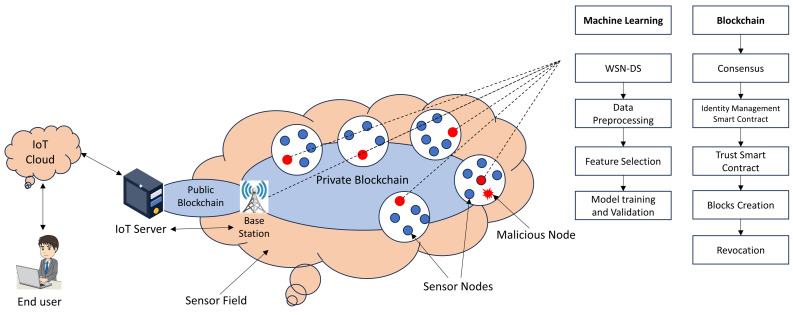
Architecture of Blockchain and machine learning integration in WSNs.

Figure 18 depicts the proposed BC-ML integrated system shows an ML detection model that can detect harmful node behaviour by examining nearby data. With the use of transfer learning, the ML model may use deviations from expected behaviour to identify potential risks. In addition, a BC-based preventive mechanism provides security against rogue nodes’ efforts to modify data without authorisation. The entire ML detection procedure is securely recorded in the BC ledger [80] to preserve its integrity. Smart contracts ensure identity management by prohibiting unauthorised access and robbing privileges in the event of harmful behaviour. A trust-based smart contract, particularly in cluster-based architectures, ensures end-to-end trust across communication nodes, reducing the impact of assaults on the impacted network segment. These smart contracts’ usefulness is increased by the ability to host ML models. While it has been suggested that ML models be used to identify susceptible smart contracts used by malicious nodes or attacks based on smart contracts, it is important to note that smart contracts may have difficulty with high-computation ML jobs. The envisioned BC topology includes public BCs linking the base station (BS) and IoT cloud in a multi-layer or hybrid architecture, along with private BCs for internal authentication. This strategy tries to handle the complex security needs of WSNs.

This section examined how combining machine learnng and Blockchain may significantly improve the security framework for WSNs. A significant conclusion is that machine learnng enables real-time threat detection, whereas Blockchain provides safe, tamper-proof data management. This dual strategy provides a comprehensive solution for minimising cyber risks in WSNs, paving the way for future advances in secure network architectures.

## 6. Limitations and Challenges

While machine learning (ML) has enormous potential for WSNs, it is critical to acknowledge that its implementation is not without problems and constraints. These limits, while surmountable, need careful consideration in the goal of efficiently implementing ML in WSNs. One significant shortcoming of ML approaches in WSNs is their inability to produce rapid, pinpoint-accurate predictions. To discover patterns and make educated judgements, ML algorithms naturally rely on the acquisition and interpretation of past data. As a result, the system’s effectiveness is heavily dependent on the number and quality of historical data at its disposal. When working with enormous datasets, however, a quandary arises since the computing expense increases in lockstep with data size. The interaction of WSN energy restrictions and the deep computational difficulties of ML algorithms needs strategic thought. One potential option is to centralise ML algorithms, which can reduce the energy cost while retaining ML’s analytical power [131].

Another issue occurs in the field of real-time validation. The predictions made by ML algorithms are vital, but proving their correctness in a real-time operating setting may be time-consuming. The inherent dynamic of WSNs, along with the necessity for rapid decision-making, necessitates the seamless integration of ML predictions and real-time execution. Obtaining this synchronisation is a significant task. Furthermore, determining the best ML approach to solve specific challenges in WSNs might be a difficult conundrum. The ML landscape is rich with algorithms, each specialised to a certain problem area. Navigating this complexity in order to find the best ML solution that matches the particular requirements and subtleties of WSNs can be a daunting endeavour [132].

Given these limitations, it is clear that while ML has significant potential for WSNs, there is an urgent need for creative methods to align its computational needs with WSNs’ energy limits and to enable smooth real-time validation. Furthermore, optimising the process of picking the best ML approaches for specific WSNs difficulties is an area that needs more inquiry and development. Addressing these challenges would surely open the way for the efficient use of ML in WSNs, allowing them to reach their full potential in a variety of applications [133].

- Localisation Challenges: In the world of WSNs, effective Path Planning for mobile sensor nodes, particularly beacon nodes, is critical. Surprisingly, there are no well-defined Path Planning algorithms targeted to movable anchor nodes. In this context, the use of machine learning (ML) appears to be a viable route, with the ability to give optimised Path Planning for individual anchor nodes in sensor networks. This holds the prospect of improving localisation accuracy while reducing energy usage, which is crucial in resource-constrained WSNs. It is important to note that numerous live applications extend their data collection points into the space of three dimensions. However, the bulk of known localisation techniques have been studied primarily in two-dimensional space. The development of localisation algorithms that are specifically tailored to three-dimensional environments, including both static and mobile WSNs, is thus urgently needed [134].
- Coverage and Connectivity Challenges: Recent efforts have made significant progress in tackling coverage and connection difficulties inside WSNs. Nonetheless, a variety of unexplored problems and difficulties remain, awaiting research. Among these difficulties is anticipating the minimal number of sensor nodes required to efficiently cover a particular region or goal, as well as finding the appropriate placement of these sensor nodes [135]. Furthermore, random Sensor Node Deployment, which is typical in real-time WSN applications, poses the possibility of coverage gaps, commonly known as “coverage holes”. Detecting and correcting such coverage gaps is a hard job, made more difficult by the dynamic nature of networks, where coverage gaps can occur spontaneously. Importantly, whereas two-dimensional coverage algorithms have made great progress, the area of three-dimensional coverage, characterised by maximum computing efficiency, remains completely unexplored.
- Anomaly Detection Challenges: The identification of anomalies is one of the most significant research challenges in WSNs. In recent years, academics have worked hard to develop a variety of strategies for detecting abnormalities. Anomalies in WSNs have far-reaching consequences, affecting communication overhead, transmission delays, and possibly sensor node data. While anomaly detection has received a lot of attention, it is only one aspect of the problem. When an abnormality is discovered, further research efforts must focus on determining the best course of action. This entails defining the necessary actions to reduce harm and maintain network integrity. It is critical to recognise that anomaly detection algorithms are intrinsically application-specific, demanding a careful selection procedure, particularly in diverse WSNs. To fit with the different needs and operating dynamics of such networks, the selected detection algorithm must strike a compromise between precision and speed [136].
- Congestion Control and Avoidance Challenges: Congestion control and avoidance are fundamental concerns in the ever-changing WSN ecosystem. When considering the multifarious nature of WSN operations, which are prone to internal disturbances and external stimuli that may result in data loss, the necessity for strong and durable congestion management systems becomes clear. The intrinsic limits of WSNs, which include limited energy and memory resources, highlight the significance of simplified congestion control at the node level, in conjunction with prudent regulation of transmission rates between nodes. Efficient congestion control necessitates an adaptive, self-learning solution capable of dynamically responding to network disturbances. This flexibility should include the ability to add or remove nodes when congestion patterns emerge throughout the network. Furthermore, there is a pressing need for novel traffic prediction techniques capable of detecting and mitigating dynamic route modifications in order to avoid congestion problems. Furthermore, further research into effective mobile agent-based data gathering systems as an alternative to traditional inter-node data transfer is warranted [137].
- Quality of Service (QoS) Challenges: A multidimensional problem occurs in the quest for Quality of Service (QoS) in WSNs. The varying needs of users and applications need the development of customised QoS standards that account for differences in sensor types, data rates, traffic management strategies, and data formats. The customizable nature of QoS standards offers a significant issue since it involves the creation of efficient cross-layer protocols capable of upgrading QoS to satisfy a wide range of requirements. The issue of developing and adhering to QoS standards appears as a complicated difficulty within the context of large-scale, heterogeneous mobile sensor networks, necessitating thorough thought and novel solutions [138].
- Integration Performance: The successful integration of Blockchain (BC) with machine learnng (ML) depends on the enhancement of each technology’s unique capabilities. For instance, data manipulation may cause ML models to perform worse, which BC can help to prevent. In order to prevent manipulation, BC guarantees [139] the integrity of the transactions used to train ML models and the recorded judgements in attack classification. Authorised nodes have access to these records for auditing and review, which helps to improve subsequent ML detection judgements. Due to this synergy, incremental ML models can more effectively identify new assaults and evolve over time to accommodate dynamically changing networks.
- Scalability: In the context of WSNs, scalability refers to network capacity, including the quantity of nodes and transactions. Scalability is highly impacted by the consensus technique and the BC type chosen. For instance, compared to Proof-of-Work (PoW), consensus algorithms like Practical Byzantine Fault Tolerance (PBFT) and Proof-of-Authentication (PoA) might increase transaction throughput. Scalability becomes difficult as data and network size grow. For better scalability, solutions include hybrid BCs with public and private BCs, as well as limiting transaction volume and ledger size. For private BCs and cooperative WSNs, voting [140] or multiparty consensus are appropriate. PoAh is a possible option for networks with limited resources.
- Lightweight Schemes: Lightweight schemes are necessary for effective BC-ML [141] integration because they maintain security while having the least amount of overhead. The storage, processing, and communication requirements for components like trust, authentication, access control, smart contracts, and consensus processes should be met. For instance, IPFS can lower data storage costs in WSNs, but communication overhead must be taken into account. The preferable consensus mechanisms requiring less computation and latency are PBFT and PoA.
- Vulnerability: While combining [142] ML and BC improves security, not all attacks are completely eliminated. Even when data are protected by BC, it is possible for them to be altered before being securely recorded in the ledger.
- Managing Network Resources: Limited-resource sensor nodes present technical difficulties, particularly in the areas of encryption, trust, authentication, and transaction validation. Due to the exponential expansion of the ledger, memory space may become limited. Solutions [143] include reducing the complexity of BC encryption, calculating hash functions more efficiently, using nodes specifically designed to store ledgers (such as CHs and BS nodes), and transferring old data to external storage or the IoT cloud. These methods seek to solve storage and processing problems while consuming less power.

## 7. Conclusions and Future Research

WSNs have become vital instruments for a wide range of applications, from data collection to environmental monitoring, because of their ease of use, specialised capabilities, and low cost. However, the broad usage of WSNs has created a slew of issues, the most pressing of which is security. Traditional security methods are impracticable due to the inherent constraints of WSNs, which include limited processing capacity and energy resources. Innovative ways are required to properly handle these security problems. We have presented a complete description of the WSN infrastructure, its operational environment, many applications, and the associated security problems in this article. To improve security, we have focused on the interaction of machine learning (ML) and WSNs. We investigated numerous ML algorithms and their applications in WSN security, shining light on both their advantages and disadvantages.

Although it is clear that ML offers intriguing prospects for enhancing security in WSNs, its deployment is not without hurdles. As we move forward, it is critical to investigate novel solutions that capitalise on the promise of ML algorithms while minimising the related complexity. This article has highlighted the importance of future research efforts to successfully utilise ML algorithms in the field of WSN security. As the WSN environment evolves, employing ML will be critical to guaranteeing the integrity, secrecy, and resilience of these networks in an increasingly linked world.

## Figures and Tables

**Table 1:** Summary of research contributions.

Reference	Approach	Contributions/Findings
Eljakani et al., 2022 [1]	• Deep learning approaches for WSN performance prediction • CNN-based MAC protocol identification MLP for PDR and SNR prediction	• Survey of recent DL methods for WSN performance prediction • Application of DL in multiple WSN layers • Exploration of cross-layer approaches • Identification of challenges in data availability and multi-parameter prediction
Rawat et al., 2014 [4]	• Overview of WSN technologies • Challenges in WSN applications • Collaborative nature of WSNs • Highlights infrastructure challenges • Importance of intelligent collaboration among nodes • No explicit discussion of limitations • Acknowledgment of resource constraints and communication reliability challenges	• Comprehensive overview of recent WSN developments • Identification of challenges and potential applications • Emphasis on intelligent collaboration and energy efficiency • Discussion of sensor node limitations • Importance of intelligent collaboration among nodes • No explicit discussion of limitations • Acknowledgment of resource constraints and communication reliability challenges • Limited focus on machine learning or DL techniques
Alsheikh et al., 2014 [6]	• Literature review of ML methods in WSNs • Text representation methods for system calls • Performance evaluation and comparison	• Identification of best algorithm for detection • Insights into ML performance in intrusion detection • Evaluation of classical ML classifiers • Benchmarking DNNs for intrusion detection
Vinayakumar et al., 2019 [8]	• Intrusion detection using DNNs • Evaluation of classical classifiers • Text representation methods for system calls • Need for systematic dataset updates • No discussion of scalability in real-world implementation	• Evaluation of DNNs and classical classifiers for intrusion detection • Proposal of Scale-Hybrid-IDS-Alert Net (SHIA) • Identification of best algorithm for detection • Insights into ML techniques for intrusion detection • Performance evaluation and benchmarking
Ahmad et al., 2022 [10]	• Survey of ML algorithms for WSN security • Discussion of security requirements in WSNs	• Identification of security requirements and challenges • Statistical analysis of security implementations • Highlighting open issues in ML adaptation to sensor capabilities • Specific ML algorithm analysis lacking • Performance evaluation not discussed
Khashan et al., 2021 [11]	• Introduction of FlexCrypt scheme for secure and efficient WSNs • Dynamic clustering for mobility support • Performance evaluation in simulation	• Introduction of FlexCrypt scheme for secure and efficient WSNs • Flexible lightweight cryptographic method • Security analysis and performance evaluation • Highlighting advantages over other ciphers
Mohan et al., 2014 [12]	• Cross-layer framework based on IEEE 802.15.4 [13]/ZigBee standards • Simulation using GloMoSim • Beacon-enabled peer-to-peer topology • LEACH routing protocol • Superframe structure utilisation • Sleep and ACK modes	• Improved energy efficiency, network lifetime, latency • Higher packet delivery ratio, lower delay, increased throughput • Reduced energy consumption
Zhang et al., 2022 [14]	• QoS model based on routing, clustering, and data fusion • Evaluation of an optimal fertilisation system	• Better energy balance and cluster head distribution • Improved energy consumption and flexibility • Insight into sensor node diagnostics • Effective fertilisation support system • Comparison of diagnostic models • Assessment of node consumption
Ramasamy et al., 2017 [15]	• Categorisation of factors for energy efficient MWSNs • Discussion of mobile sensor node architecture • Exploration of various aspects of MWSNs	• Insights into mobile sensor node architecture • Exploration of MWSN aspects
Ali et al., 2017 [16]	• Survey of real-time WSN applications • Mention of sensor technology advancements	• Highlighting sensor technology advancements • Lack of specific quantitative results • No exploration of scalability, interoperability, security, or cost-effectiveness
Phoemphon et al., 2018 [17]	• Hybrid model: Fuzzy Logic + ELM + PSO • Centroid method with weight (wi)	• Improved WSN localisation accuracy • Integration of soft computing techniques • Lack of discussion on scalability and energy efficiency • No analysis of network topology impact • Fuzzy logic limitations not addressed • Limited comparison with other localisation techniques

**Table 2:** Comparison of wireless Communication Protocols.

Protocol Characteristics	Wi-Fi (IEEE 801.11n [12])	ZigBee (IEEE 802.15.4 [13])	6LoWPAN (IETF RFC-6282 [24])	Bluetooth (IEEE 802.15.1 [25])	BLE (IEEE 802.15.1 [25])	Z-Wave (ISO/IEC 14543 [26])
Frequency Range	2.4–5 GHz, 120 kHz	2.4 GHz, 868/915 MHz	868/921 Hz, 2.4–5 GHz	2.402–2.482 GHz	2.402–2.482 GHz	2.4 GHz, 868/915 MHz
Max Data Rate	11–54 Mbps	20/40 kbps; 250 kbps	10–40 kbps, 250 kbps	0.7–2.1 Mbps	0.27 Mbps	9.6–40 kbps
Effective Range	10–100 m	10–1000 m	10–100 m	15–20 m	10–15 m	30–50 m
Modulation Schemes	BPSK, QPSK, OFDM, M-QAM	D-BPSK, OQPSK, QPSK	BPSK, O-QPSK, ASK	GFSK, CPFSK, 8-DPSK	GFSK	FSK, GFSK, Narrowband
Network Topology	Star, Tree, Point-to-Point	Star, Mesh, Cluster Tree	Star, Mesh, Point-to-Point	Star	Star, Mesh, Point-to-Point	Mesh
Network Size	Up to 32 devices	Up to 65536 nodes	Up to 100 nodes	Up to 8 devices	Not Applicable	Up to 232 nodes
Encryption	RC4 Stream, AES Block Cipher	128-bit AES	128-bit AES	AES-CCM	128-bit AES	128-bit AES
Coding Techniques	MC-DSSS, CCK, OFDM	DSSS (1–15), DSSS (4–32)	Header Compression, DSSS	FSK, GFSK, RISS	Adaptive CCK	Manchester, NRZ
Channel Bandwidth	20–25 MHz, 0.3/0.6 MHz	0 MHz (fixed), 2–5 MHz	Not Applicable	8 MHz	2–5 MHz	Fixed (1 MHz)

**Table 3:** Overview of machine learning techniques for enhancing WSN performance and security.

Reference	WSN Category	Approach	Evaluation Parameter	Inference
Phoemphon et al., 2018 [17]	Localisation, Determining the relationships between anchor nodes and their locations using Received Signal Strength Indicators (RSSIs)	Fuzzy Logic and Extreme Learning Machine (ELM) with Vector Particle Swarm Optimisation (PSO), SVM-RBF	Location estimation precision, node density, sensing coverage	Enhances the location approximation in WSNs, and the ELM-based technique is found to be superior to other techniques such as Fuzzy Logic, Genetic Algorithms (GAs), Neural Networks (NNs), and SVMs
Gharghan et al., 2016 [57]	Localisation, distance estimation between mobile sensor node and anchor node in outdoor and indoor environments	PSO-ANN, Levenberg–Marquardt (LM) training algorithm	Mean absolute error	The hybrid PSO-ANN algorithm significantly improved the distance estimation accuracy compared to the traditional LNSM method
Bernas et al., 2015 [58]	Localisation	FCNNs	Arithmetic mean of error	Use of FCNNs and signal strength clustering helps in reducing the memory required to store the received signal strength map
Banihashemian et al., 2018 [59]	Localisation	Neural networks	Localisation accuracy and storage overhead	Lower localisation error rate and requires less storage compared to analogous methods in different environmental conditions
El Assaf et al., 2016 [60]	Localisation, Distance estimation and power-efficient correction mechanisms	ANNs	Accuracy and robustness against anisotropic signal attenuation	Proposed algorithm significantly outperforms most representative range-free localisation algorithms in terms of accuracy and robustness against anisotropic attenuation
Xie et al., 2013 [61]	Anomaly Detection	K-Nearest Neighbour (k-NN)	Accuracy	Proposed scheme is effective and robust for online anomaly detection in WSNs
Wazid et al., 2016 [62]	Anomaly Detection, detect two types of malicious nodes: blackhole and misdirection nodes	K-Means Clustering	Detection Rate	Proposed technique using K-means clustering achieves significantly better results compared to existing related schemes for detecting malicious nodes in WSNs
Javaid et al., 2019 [63]	Error Detection	Enhanced K-Nearest Neighbour (Ek-NN), Enhanced Extreme Learning Machine (EELM), Enhanced Support Vector Machine (ESVM), and Enhanced Recurrent Extreme Learning Machine (ERELM)	Detection Accuracy (DA), True Positive Rate (TPR), and Error Rate (ER)	Proposed methods outperform existing techniques and provide better results for belief function fusion and fault detection in WSNs
Emperuman et al., 2020 [64]	Error Detection	Continuous Density Hidden Markov Model (CDHMM) and various neural networks (NNs)	Detection accuracy, false positive rate, F1-score, and the Matthews correlation coefficient	Learning vector quantisation NN classifier outperforms the other classifiers in terms of detection accuracy rate
Sun et al., 2019 [65]	Error Detection	Recursive Principal Component Analysis (RPCA) model and Support Vector Data Description (SVDD)	Sample evaluation criterion rF(i)	Proposed algorithm in the research paper can satisfy the real-time needs of data stream processing and track data changes well. It improves the safety factor of monitoring sites and allows for timely repair or replacement of faulty nodes.
Vasuhi et al., 2016 [66]	Target Tracking	Interactive Target Tracking in Wireless Sensor Networks (ITTWSN)	Accuracy	Proposed ITTWSN scheme using Interactive Multiple Model (IMM) is more accurate in target tracking compared to the existing Kalman Filter (KF) based approach in a WSNs environment
Zhou et al., 2014 [67]	Target Tracking	Variational Bayesian Filtering (VBF)	Accuracy	Focuses on the convergence properties of the proposed VBF scheme for target position estimation in WSNs
Xue et al., 2017 [68]	Target Tracking, sparse signal recovery in WSNs for the detection of sparse events and localisation purposes	Sparse Bayesian Learning (SBL)	Accuracy	Proposed approaches in the research paper lead to superior performance compared to reference methods in terms of sparse signal recovery in WSNs
Das et al., 2018 [69]	Authentication	Long short-term memory (LSTM)	Classification accuracy	Deep learning classifier shows high resilience to advanced software radio adversaries
Liao et al., 2019 [70]	Authentication	Counter Propagation Neural Network (CPNN), Deep Neural Network (DNN)	Accuracy	Proposed DL-based PHY-layer authentication method can implement lightweight authentication for the sensor nodes under the edge computing (EC) system in Industrial WSNs
Ashibani et al., 2020 [71]	Authentication	Random Forest, K-Nearest Neighbours, Gradient Boosting Classifier, and Multi-Layer Perceptron	F-Measure	Presented model authenticates users based on application access events with high F-measure
Rezaee et al., 2018 [72]	Congestion Control	Fuzzy Proportional-Integral-Derivative (PID)	Data loss rate and end-to-end delay	Proposed protocol outperformed other approaches in terms of data loss rate and end-to-end delay, based on simulation results using the OPNET simulator and MATLAB
O’Mahony et al., 2020 [73]	Congestion Control, Safety-critical applications	Support Vector Machine (SVM) and Random Forest	Classification accuracy	Investigates supervised machine learning techniques for channel identification in WSNs using SVM and Random Forest algorithms
Alsheikh et al., 2016 [74]	Congestion Control	Autoencoder networks	Evaluated using real-world datasets and compared with conventional methods for temporal and spatial data compression	Proposed algorithm outperforms several existing WSN data compression methods in terms of compression efficiency and signal reconstruction
Gholipour et al., 2019 [75]	Congestion Control	Support Vector Machines (SVM)	Energy consumption, packet loss, end-to-end delay, throughput, and network lifetime	Proposed method using SVM and genetic algorithm tuning improves network throughput, decreases energy consumption, packet loss, and end-to-end delay in WSNs. It also significantly improves network lifetime under different traffic conditions, especially in heavy-traffic areas
Sun et al., 2017 [76]	QoS, monitoring and control in smart grids	Wavelet-Neural-Network-based Link Quality Estimation (WNN-LQE)	Accuracy	Presents comparative experimental results to demonstrate the validity of the proposed LQE algorithm
Lee et al., 2016 [77]	QoS	RescueNet	Accuracy	Introduces a self-adaptation framework for the network called RescueNet to enhance QoS in disaster scenarios
Renold et al., 2017 [78]	QoS	Multi-Robot Learning (MRL)	Packet delivery ratio, end-to-end delay, path stability, throughput	Proposed MRL algorithm achieves stabilised performance in terms of packet delivery ratio, end-to-end delay, path stability, and throughput in underwater sensor networks (UWSNs)
Revanesh et al., 2021 [79]	Malicious Attacks	Deep Convolutional Neural Networks (DCNN), Simulated Annealing-based Search Optimisation (SSO)	Latency, Energy, Computational cost, and Accuracy	Proposed method shows a 97% improvement in energy efficiency and a 90.5% improvement in computational cost, with better accuracy and latency compared to existing systems
Zhao et al., 2019 [80]	Cyber Attacks	Decision trees, Random forests, Gradient boosting, and XGBoost with Hierarchical Ensemble Transfer Learning (HeTL) and Centralised Ensemble Hierarchical Transfer Learning (CeHTL)	Accuracy	Proposed hierarchical ensemble transfer learning methods significantly improve the performance of individual base learners and the overall system in terms of accuracy

**Table 4:** Machine-learning-based enhancements for Quality of Service (QoS) in WSNs.

ML Approach	Relevant Research	Computational Complexity	Targeted Goals
Artificial Neural Network (ANN)	[76]	Minimal	Assessment of Link Quality
	[96]	Moderate	Detection of Faulty Nodes
	[119]	Reasonable	Fusion of Data and Energy Efficiency
Reinforcement Learning	[77]	Moderate	Cross-Layer Communication Framework
	[78]	Minimal	Control of Network Topology and Data Distribution Protocol
	[120]	Substantial	Composition of Services with Fulfillment of Constraints
	[121]	Moderate	Adaptive Cooperative Routing in a Distributed Manner

## Data Availability

Not applicable.
